# Efferocytosis by macrophages in physiological and pathological conditions: regulatory pathways and molecular mechanisms

**DOI:** 10.3389/fimmu.2024.1275203

**Published:** 2024-05-08

**Authors:** Yan−Ran Sheng, Wen−Ting Hu, Siman Chen, Xiao−Yong Zhu

**Affiliations:** ^1^ Obstetrics and Gynecology Hospital, Fudan University, Shanghai, China; ^2^ Key Laboratory of Reproduction Regulation of NPFPC, SIPPR, IRD, Fudan University, Shanghai, China; ^3^ Shanghai Key Laboratory of Female Reproductive Endocrine Related Diseases, Fudan University, Shanghai, China

**Keywords:** efferocytosis, macrophages, metabolic reprogramming, molecular mechanisms, combination therapy

## Abstract

Efferocytosis is defined as the highly effective phagocytic removal of apoptotic cells (ACs) by professional or non-professional phagocytes. Tissue-resident professional phagocytes (“efferocytes”), such as macrophages, have high phagocytic capacity and are crucial to resolve inflammation and aid in homeostasis. Recently, numerous exciting discoveries have revealed divergent (and even diametrically opposite) findings regarding metabolic immune reprogramming associated with efferocytosis by macrophages. In this review, we highlight the key metabolites involved in the three phases of efferocytosis and immune reprogramming of macrophages under physiological and pathological conditions. The next decade is expected to yield further breakthroughs in the regulatory pathways and molecular mechanisms connecting immunological outcomes to metabolic cues as well as avenues for “personalized” therapeutic intervention.

## 1 Introduction

**Figure 1:**
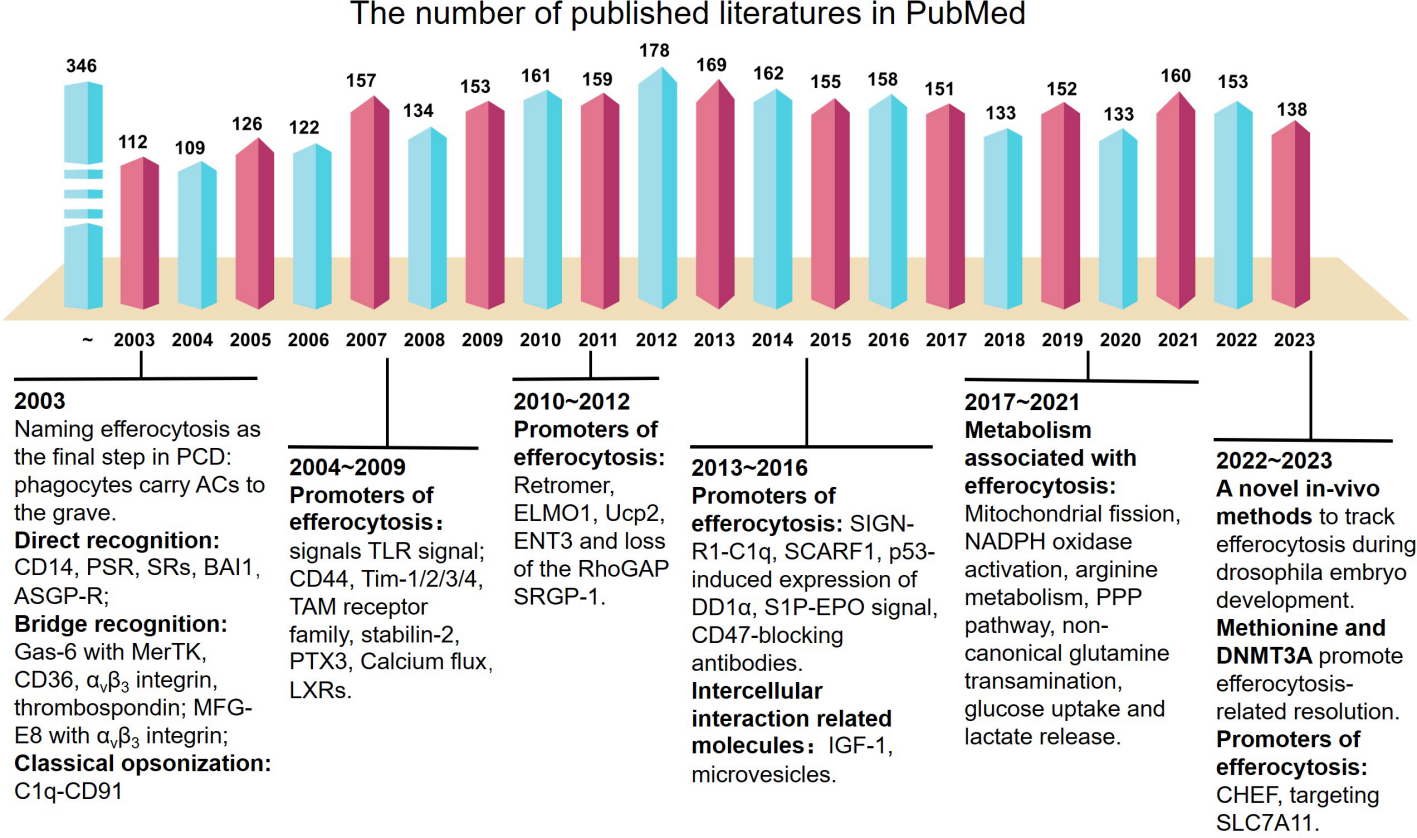
Key breakthroughs and milestones in the literature on efferocytosis since 2003. The literatures shown in PubMed each year is marked out, along with the exciting and interesting discoveries related to efferocytosis. More detailed information is shown here: https://pubmed.ncbi.nlm.nih.gov/?term=%28clearance+of+apoptotic+cells%29+OR+%28efferocyotsis%29&timeline=expanded&sort=date&sort_order=asc. PCD, programmed cell death; TLR, toll-like receptors; TAM receptor family, tyrosine kinase Tyro3, Axl, and Mer; PTX3, Pentraxin 3; LXRs, Liver X receptors; ENT3, equilibrative nucleoside transporter 3; SIGN-R1, a C-type lectin; SCARF1, scavenger receptor F1; DD1α, Death Domain1α; S1P, sphingosine 1-phosphate; IGF-1, insulin-like growth factor 1; EPO, erythropoietin; NADPH, NAD phosphate; PPP pathway, pentose phosphate pathway; DNMT3A, DNA methyltransferase-3A; CHEF, chimeric receptor for efferocytosis; PSR, phosphatidylserine receptor; MFG-E8, milk fat globule–epidermal growth factor 8; SRs, scavenger receptor superfamily; ASGP-R, asialoglycoprotein receptor.

Over the past two decades, much has been deciphered regarding the phagocytosis of apoptotic cells (ACs) (1, 2). In 2003, a ubiquitous process of removing numerous ACs in multicellular organisms daily, along with the term “efferocytosis”, was suggested (**Figure 1**). Efferocytosis emerges from the final step of apoptosis (3), which occurs rapidly to prevent secondary necrosis and the release of proinflammatory moieties and antigenic cell components. Moreover, elimination of apoptotic or dying cells appears to be a widespread biological process with highly conserved mechanisms and specific signaling pathways (4).

**Figure 2:**
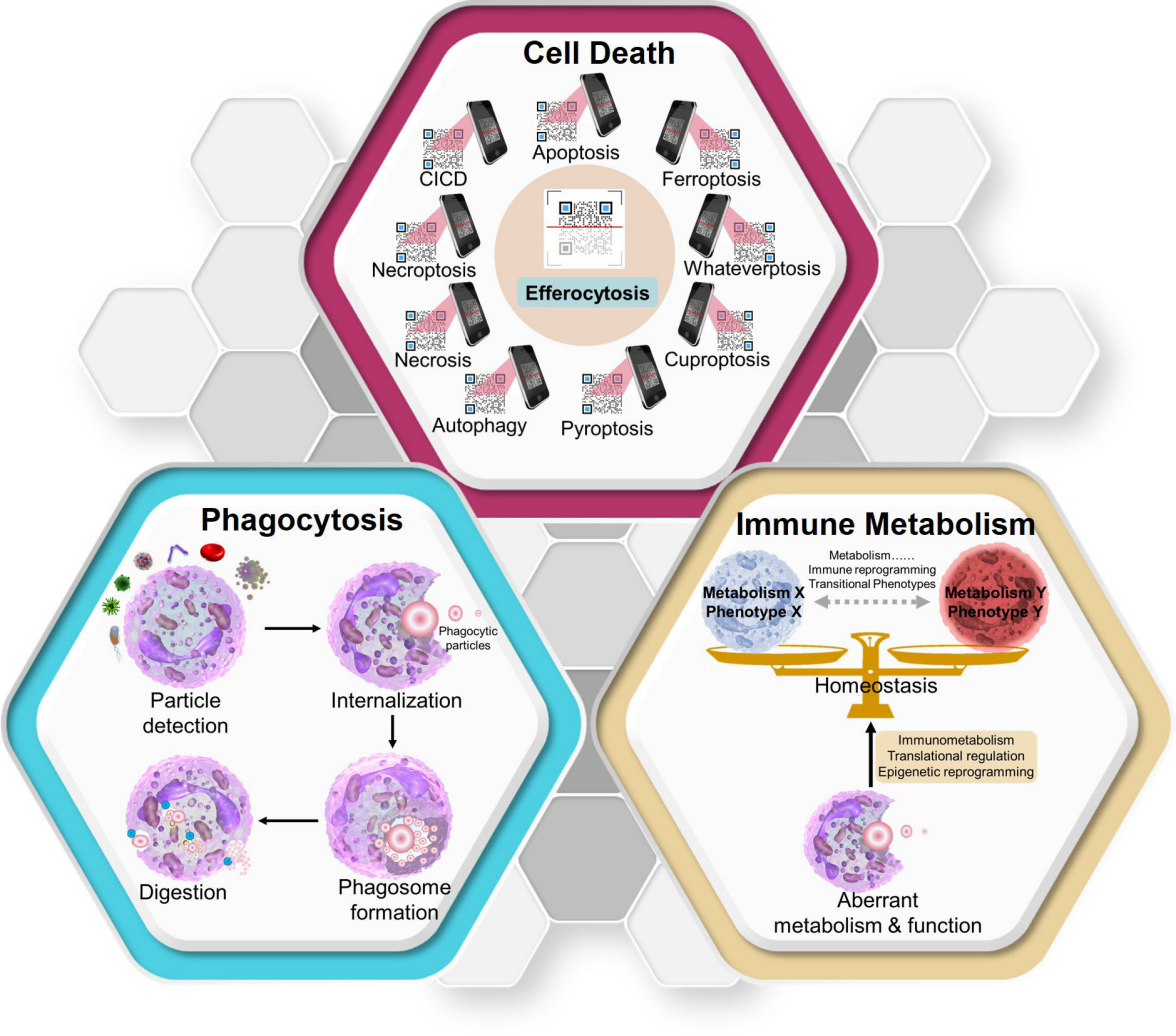
Three key concepts that aid in the clear understanding of efferocytosis. Diverse modes of cell death have different effects on efferocytosis. Efferocytosis carried out by macrophages can be used to identify the death mode of “swallowed cargos” (like scanning a QR code) and make different responses (though the specific mechanisms need further research). As a form of phagocytosis, efferocytosis can also be divided into four periods. After degradation of swallowed materials, macrophages maintain homeostasis through metabolic immune reprogramming. CICD, caspase-independent cell death. Whateverptosis: the types of cell death remaining to be discovered in the future.

Three major research areas have been emphasized to provide foundational understanding of efferocytosis—(i) phagocytosis (universal immune–biologic process), (ii) the mechanisms orchestrating different types of cell death and its consequences (5), and (iii) the immune metabolism (6) influencing “macrophage programming” (**Figure 2**).

The past century has witnessed evidence of the uptake of various types of particles by different types of phagocytes. These particles arise from simple and complex organisms, and phagocytes include professional (e.g., macrophages) and non-professional (e.g., epithelial and endothelial cells) types. Phagocytosis involves the ingestion and elimination of particles >0.5 μm in size within a plasma membrane envelope. Phagocytosis contributes to pathogen elimination and homeostasis of the internal environment (7). Metchnikoff (1845–1916) explored the role of phagocytosis and won the Nobel Prize in 1908. In 1995, Rabinovitch coined a term for a specialized group of cells with highly efficient activity as “professional phagocytes” (8). As phagocytes, macrophages contain a high concentration of acid hydrolases that efficiently degrade ingested particles. However, some tissue-resident macrophages are poorly phagocytic despite the presence of typical macrophage-related markers on the membrane surface. Gonzalez et al. sought to provide possible explanations for this phenomenon by investigating the phagocytic properties of resident macrophages in various tissues (9). Although there are many redundant phagocytic markers on the membrane surface, intracellular processing and signal transduction are less redundant. Gonzalez et al. highlighted the importance of post-transcriptional regulation by revealing that phagocytic cells in different microenvironments share commonly regulated genes. The mechanism underlying this phenomenon warrants further investigation. Phagocytosis involves four main phases: (i) particle detection, (ii) internalization, (iii) phagosome formation, and (iv) phagosome maturation to transform it into a phagolysosome (blue box in **Figure 2**). The consequences of phagocytosis vary depending on the phagocytes, the “cargo” to be swallowed, and the subsequent regulatory mechanisms.

The type and mechanism of cell death are the cornerstones of efferocytosis. Various types of cell death have been discovered, including apoptosis (programmed cell death) (10), caspase-independent cell death (CICD) (11), autophagy (12), pyroptosis (13), cuproptosis (14), ferroptosis (15), necroptosis (16), necrosis (17), and whateverptosis (types of cell death remaining to be discovered). Different types of cell death influence the immune responses of phagocytes in various ways.

Efferocytosis is an interesting and exciting process that involves cell death, phagocytosis, and immune metabolism. During different cell death processes, dying cells can release unique macromolecules to interact with efferocytes (“find-me,” “eat-me,” and “post- engulfment”). These macromolecules function similar to a “QR code” in that macrophages and other efferocytes “scan” codes and decode information from dying cells to produce immunoreactions. Most studies highlight the repair and immunosuppression of efferocytosis; however, some studies have revealed pro-inflammatory outcomes after efferocytosis (18, 19). Therefore, the precise regulation of these processes remains unknown (red box in **Figure 2**).

Efferocytosis is considered the final step of apoptosis. Phosphatidyl serine (PS) “flipping” from the inner leaflet to the outer leaflet of ACs is the most well-studied “eat-me” signal and is identified by a range of phagocyte receptors. These receptors may be involved in the direct or bridging recognition. Direct recognition receptors include the PS receptor, cluster of differentiation (CD)14 scavenger receptor superfamily, brain-specific angiogenesis inhibitor (BAI)-1, and asialoglycoprotein receptor (ASGP-R) (20). Bridge recognition receptors include growth arrest-specific protein6 (GAS-6) with Mer-TK (tyrosine-kinase-activated receptor), CD36, α_γ_β_3_ integrin, thrombospondin, milk fat globule-epidermal growth factor 8 (MFGE8, also called lactadherin) with α_γ_β_3_ integrin, and C1q-CD91. These receptors were first described by Aimee M. deCathelineau and colleagues (1) in 2003. During 2004–2009, scientists discovered that the toll-like receptor (TLR) signal participated in phagosome maturation (21) and that CD44 (22), T cell membrane protein (Tim)1/2/3/4 (23, 24), a family of tyrosine kinases [Tyro3, Axl, and Mer (TAM receptor family)] (25, 26), stabilin-2 (27), pentraxin 3 (28), calcium flux (29), and liver X receptors (LXRs) (30) promote efferocytosis. In the next decade, the research focus shifted to the regulation of intracellular transcription factors and from epigenetics to efferocytosis. Retromer (31), cell motility protein 1 (ELMO1) (32), Ucp2 (33), equilibrative nucleoside transporter-3 (34), and the loss of RhoGAP SRGP-1 (35) were found to promote efferocytosis. Moreover, from 2013 to 2016, SIGN-R1-C1q (36), scavenger receptor class F, member 1 (SCARF1) (37), p53-induced expression of Death Domain-1α (38), sphingosine-1-phosphate (S1P) erythropoietin signal (39), and CD47-blocking antibodies (40) were found to promote efferocytosis. Insulin-like growth factor (IGF)-1 and microvesicles from macrophages dampened the uptake of larger apoptotic cells while enhancing the engulfment of microvesicles and decreasing inflammatory responses by non-professional phagocytes (41).

After recruitment and engulfment, efficient degradation and inflammatory programming are important for homeostasis and normal functioning of all types of organisms and systems. Since 2017, with the development of live cell tracking methods for efferocytosis, the metabolism of carbohydrates (42), lipids (43), free fatty acids (FAs) (44), amino acids (45) and nucleotides (46) from ACs and their effects on macrophage programming have been slowly revealed (see Section 5).

The metabolic characteristics of cancer cells are distinct from those of resting tissues. Recent studies have revealed the effect of metabolic phenotypes on the characteristics of proliferating (especially immune) cells transitioning between different states. Cancer and immune cells have numerous metabolic similarities (as well as critical differences) that affect the diagnosis and treatment strategies for immune system diseases and cancers. Traditional studies of cancer cell metabolism have suggested that glycolysis promotes immune tolerance, whereas oxidative phosphorylation (OXPHOS) promotes an anti-inflammatory immune response (47, 48). However, recent studies have revealed different and even diametrically opposite results regarding metabolic immune reprogramming associated with efferocytosis by macrophages (49–52). Geeraerts et al. revealed the metabolic heterogeneity of tumor-associated macrophages. Lactate, the product of glycolysis, differentially affects these macrophages to elicit antitumoral or protumoral effects (53). The differentiation and activation of tumor-associated macrophages also require lipid accumulation and metabolism (54). However, the role of amino acid metabolism in tumor progression and efferocytosis remains unclear (55). There is a considerable replacement of cells in the tumor microenvironment. This heterogeneity can be attributed to genetic and environmental factors (56). Genetic factors include instability and epigenetic modifications, while environmental factors include the specific spatial environment of tissues (including the infiltration of immune cells, secretion of cytokines, and angiogenesis) as well as the distribution of various metabolites. Such heterogeneity has a profound impact on clinical outcomes and response to treatment. Once ACs are ingested, macrophages are subjected to a large metabolic load. However, the contrasting metabolic roles of efferocytosis and cancer require further investigation. Moreover, the influence of efferocytic metabolism on macrophage programming is incompletely understood (yellow box in **Figure 2**).

In this review, we provide a chart of the vast literature on efferocytosis, which has increased by 150 articles per year over the last two decades (**Figure 1**). Efferocytosis occurs at the intersection of apoptosis, metabolism, and immunoregulation (**Figure 2**), and these phenomena contribute to our knowledge of efferocytosis. Here we summarize the key physiological and pathological contexts of efferocytosis and the emerging therapeutic applications used to modulate efferocytosis. Moreover, with a focus on tissue-resident macrophages (the most common professional efferocytes), we describe the latest progress in the immune metabolic mechanisms that regulate efferocytosis within this framework. Finally, we discuss key questions that will likely drive future efferocytosis studies.

## 2 Physiological functions of macrophages during efferocytosis

As the most studied phagocyte, a macrophage that can perform moderate efferocytosis plays an important role in the maintenance of homeostasis under physiological conditions. Efferocytosis can be regarded a safe method of “garbage disposal”. The internal environment of the human body is characterized by a universal turnover of cells and purging of ACs. In 1992, Dini et al. employed a rat model with fluorogenic labeling to demonstrate that clearance of apoptotic hepatocytes was mediated by ASGP-R in hepatocytes under physiological conditions, representing a sugar recognition system in the liver (20). However, the phenotypic changes that occur after this type of phagocytosis were not studied further. Another study reported that AC removal by macrophages limited the release of thromboxane-B2 (57). Gradually, researchers began to regard the apoptotic cargos as “bioactive treasures” released from dying cells, which promoted a pro-resolving macrophage phenotype (58). Efferocytosis enables anti-inflammatory and homeostatic maintenance. This pro-resolving macrophage phenotype suppresses the expression of proinflammatory cytokines and upregulation of pro-resolving mediators and angiogenic growth factors (59).

In the hippocampal dentate gyrus of the central nervous system (CNS), efferocytosis by ramified microglia (phagocytic cells that remove ACs and crops in the brain) balances cell death and neurogenesis to promote homeostasis and brain development, although the underlying mechanism requires further research (60). Paneth cells are pluripotent cells found in the small intestine. Paneth cells are the building blocks of intestinal health because they secrete antimicrobial peptides to ensure a sterile environment and efferocytosis of ACs from the small intestinal crypts (61). In the immune system, apoptotic B cells in the early germinal centers of lymphoid follicles locally activate follicular macrophages into classical tangible body macrophages for efferocytosis, which can prevent antibody-mediated autoimmune diseases (62). In mice, large peritoneal macrophages undergo efficient efferocytosis to maintain a homeostatic peritoneal microenvironment and promote self-tolerance (63). A “multi-omics” analysis of cardiac development/function from early embryo to adult mice revealed that a subpopulation of major histocompatibility complex class II-positive resident macrophages displayed arachidonic acid metabolism involved in efferocytosis, though the dysfunction of efferocytosis during this process was not clarified (64). Decidual macrophages efferocytosis is also important during pregnancy to maintain the homeostasis at the maternal–fetal interface (65). Morales et al. recently clarified the paradigm of microglial dominance in efferocytosis in the developing retina and demonstrated that intercellular interactions between Müller glia and microglia occur before efferocytosis (66).

People experience a gradual decline in physical strength with age, a process that has been intensively researched. Transient cellular senescence is beneficial for defense against various stresses; however, the accumulation of senescent cells in organs can lead to the breakdown of homeostasis, tissue deterioration, and tumorigenesis. Senescent cells were refractory to macrophage-mediated efferocytosis, and more senescent than apoptotic cells are observed in the aging body. Schloesser’s team uncovered that senescent cells are not only exempt from efferocytosis but also suppress macrophage-mediated corpse removal with the upregulation of the “do not eat me” CD47–QPCT/L axis (67). Senescent and aged macrophages exhibited defective efferocytosis that contributes to pathological inflammation (68).

In addition to macrophages, some non-professional phagocytes in certain tissues function under physiological conditions—for example, instead of macrophages, epithelial cells of the mouse mammary gland engulf apoptotic epithelial cells and clear residual milk after the cessation of lactation in C57BL/6 mice. This process favors the remodeling of breast tissue and prevents mastitis (69). Moreover, bone marrow mesenchymal stromal cells undergo efferocytosis to influence the remodeling of bone marrow and bone loss and maintain homeostasis of the bone marrow microenvironment along with bone marrow macrophages (70). In the male genitourinary system, Sertoli cells are specialized phagocytes responsible for preventing the accumulation of apoptotic germ cells in the seminiferous tubules *via* efferocytosis. Smoothelin-like 2 has been shown to regulate efferocytosis and lactate metabolism in Sertoli cells of mice to achieve a homeostatic state (71). In the visual system, some scavenger receptors have a direct role in the tight regulation of the circadian rhythm by participating in the clearance of the outer segments of photoreceptors by retinal-pigment epithelial cells (72). Although this process is not strictly efferocytosis, it does involve scavenger receptors. Therefore, one can speculate that efferocytosis may also play a role in the maintenance of circadian rhythms. The “find me” signals also attract neutrophils and efferocytosis by neutrophils has been revealed to be involved in inflammation (73, 74) and colorectal cancer (75).

Taken together, these results suggest that many physiological processes require (or are linked to) efferocytosis in multiple systems of the body.

## 3 Pathological contexts involving efferocytosis by macrophages

Considering the multiple functions of efferocytosis performed by macrophages, any insufficiency in efferocytosis facilitates tissue damage, inflammation, and disease development. Diseases may cause defective efferocytosis through various mechanisms (76, 77), as discussed below.

After damage, the CNS requires effective efferocytosis to initiate regenerative responses and rearrange the neuronal circuits (78). Alzheimer’s disease (AD) is a common neurodegenerative disorder. The genes associated with AD include apolipoprotein E, adenosine triphosphate (ATP)-binding cassette transporter A7, triggering receptor expressed on myeloid cells-2, and phospholipase C-γ-2. These genes are essential for efficient microglial efferocytosis (79). Pannexin1 (Panx1) channels allow anions and relatively small molecules (e.g., ATP) to pass through them. Panx1-mediated ATP release from ACs contributes to macrophage recruitment (80). The progression of experimental autoimmune encephalomyelitis (EAE) in mice (which manifests as multiple sclerosis in humans) is associated with Panx1 channels. The blockade or knockout of Panx1 channels in mice has been shown to delay the onset of EAE and ameliorate EAE signs (81). In addition, LXRs (82) and MerTK (83) are associated with multiple sclerosis/EAE. Recently, Panx1 channels were found to be involved in migraine, chronic headache, and epilepsy along with the development and maintenance of long-term spatial reference memory (84). The efferocytosis-related molecule BAI1 is a promising therapeutic target for CNS-related diseases (85). Duman et al. revealed the role of BAI1 in learning and memory (86). C1qa is involved in the complement cascade. It was found to be involved in epilepsy because C1q-knockout mice failed to eliminate excessive CNS synapses and presented with epileptiform activity (87). A study of European ancestry revealed that GLUP1 and efferocytosis-related pathway were associated with schizophrenia (88).

The efferocytic receptors β_2_ integrins and MerTK are involved in autoimmune uveitis (89) and retinal degeneration (83), respectively. The deletion of certain efferocytosis components in retinal-pigment epithelial cells leads to specific damage to the retinal integrity (90). Dysfunction of receptors for advanced glycation end products (RAGE) leads to lung fibrosis and allergic airway inflammation (91). In addition, the platelet P2Y12 receptor (92), a low-molecular-weight guanosine triphosphate (GTP) belonging to the Rho family RAC1 (93), and MerTK (83) have been shown to be involved in allergic airway inflammation. The fatty acid transporter CD36 facilitates phosphorylation of the transient receptor potential vanilloid-4 and inhibits hydrogen peroxide-mediated lung injury (94). Macrophages perform efferocytosis through cross-talk with non-professional phagocytes (e.g., airway epithelial cells) to control tissue inflammation through IGF-1 (41). In a recent study, interstitial macrophages rather than alveolar macrophages were found to clear apoptotic alveolar type 2 epithelial cells from the lungs during influenza infection (66). However, the underlying mechanism requires further elucidation.

In the urogenital system, a protein within the cytoplasm, ELMO1, connects the efferocytosis receptor BAI1 and RAC1 to perform engulfment. Therefore, dysfunction of ELMO1 can result in testicular disease and diabetic nephropathy (32). MerTK dysfunction has been shown to be associated with reduced fertility (83). In addition, the bridge protein GAS6 participates in efferocytosis by interacting with the TAM family, while abnormal GAS6 expression is associated with nephritis (95).

Several studies have reported the relationship between efferocytosis and atherosclerosis. Many apoptotic leukocytes reside in atherosclerotic plaques. Macrophages efficiently undergo efferocytosis during lesion formation. MFGE8 has been identified as an important player in attenuating inflammation *via* efferocytosis (96). A meta-analysis revealed that the MFGE8 variants rs534125149 and rs201988637 independently protected against atherosclerosis. Therefore, the inhibition of MFGE8 expression may reduce the risk of atherosclerosis (97, 98). The low-density lipoprotein receptor-related protein (LRP1) (99), C1qa (100, 101), myeloid-specific glucose transporter (GLUT)1, LXRα/β, peroxisome proliferator-activated receptors (PPARs) (102), and the GTPase dynamin-related protein-1 (103) have also been shown to be involved in atherosclerosis by being present in the different stages of efferocytosis (104). In addition, vascular endothelial growth factor-C from macrophages performing efferocytosis ameliorates ischemia–reperfusion injury and inflammation (105). The BAI1 ELMO1 RAC1 pathway is triggered to maintain cholesterol balance after efferocytosis by macrophages, and dysfunction of this signal might lead to dyslipidemia (106). Moreover, legumain (Lgmn) released from cardiac-resident macrophages promotes cardiac repair after myocardial infarction by improving efferocytosis (107).

Efferocytosis-related molecules such as G protein-coupled receptor G2A (108), CD300 family member CD300f (109), integrins (110), and RAC1 (111) have been shown to be linked to inflammatory bowel disease/colitis. CD36 is also linked to diet-induced obesity (112, 113). Thus, MFGE8 is considered a promising treatment for type 1 diabetes mellitus, inflammatory bowel disease, or colitis (96). BAI1 is expressed by gastric phagocytes and mediates efferocytosis to induce anti-inflammatory effects and cure gastritis (114). However, the paradoxical role of MerTK in colon cancer remains unclear (78, 115). After efferocytosis, macrophages release pro-resolving factors that promote tissue repair in inflammatory bowel disease (116).

Insufficient efferocytosis is a major contributor to systemic lupus erythematosus (SLE). Indeed patients with SLE often demonstrate defective efferocytosis and AC accumulation (117). G2A (118), CD300f (109, 119), integrins (89), protein S (120), and SCARF1 (37, 121) are involved in the etiology of autoimmunity accompanied by aberrant efferocytosis in macrophages. Multiple efferocytosis-related molecules, such as S1P (122), Tim4 (123), MerTK (124), C1qa (125), PPARs (126), and ATP-binding cassette transporter A1 (ABCA1) (127), are associated with SLE. Disintegrin and metalloproteinase domain-containing protein (ADAM)10 and ADAM17 have been found to reduce efferocytosis efficiency by cleaving PS receptors on the AC surface. ADAM10/17 cleavage activity is particularly high in SLE models (128, 129) and juvenile patients with SLE (130). The well-known efferocytosis-related receptors MFGE8 (131, 132) and LXRα/β (82) are involved in autoimmunity and SLE. RAGE is regarded as a target for treating sepsis because of its role in activating inflammatory signals (133–135). In addition, in a mouse model of hepatic graft-*versus*-host disease, GAS6^-/-^ mice demonstrated a higher transplantation success rate than wild-type mice (136).

Efferocytosis by macrophages has been reported in wound healing (including in patients with diabetes mellitus), tissue regeneration, and tissue development in muscles, skin, and joints (137), with an increased requirement for fatty acid oxidation and the electron transport chain (59). RAGE has been found to have a critical role in muscle regeneration (138, 139) and melanoma (140), while GAS6is is considered a therapeutic target for melanoma (141). The angiogenic function of C1qa has also been emphasized in wound healing (142). Mice lacking DNase II have been shown to exhibit symptoms of chronic polyarthritis (akin to rheumatoid arthritis in humans) (143). In addition, the therapeutic potentials of MerTK (144) and RAC1 (145) against arthritis have been uncovered. The administration of low-dose aspirin has been shown to improve cutaneous wound healing by reprogramming efferocytotic macrophages in a mouse model of DM (146).

These studies suggest that efferocytosis promotes tissue repair and the resolution of inflammation. Most of these studies have shown that deficiencies in efferocytosis-related molecules/receptors promote a disease state, whereas relatively few studies hold the opposite opinion. Scholars tend to study the mechanisms after successful modeling; however, as each disease is dynamic, different findings may be observed during different stages of the disease. Therefore, it is necessary to study the dynamic changes in efferocytosis during the course of disease.

## 4 Therapeutic applications of efferocytosis

Abnormal efferocytosis contributes to several human disorders, and efforts to exploit selective targets on the sophisticated machinery of efferocytosis have been ongoing for decades. Previously, a reduction in inflammation and treatment of autoimmune diseases were achieved mainly by improving apoptosis or regulating phagocytotic ability (147, 148). Several approaches have been used to enhance phagocytosis in mice. Macrophages with chimeric antigen receptors have been shown to have efficient antigen-specific phagocytic ability, reduce tumor burden, and prolong overall survival in two mouse models of solid tumor xenografts (149). The helix B surface peptide has been shown to increase the phagocytic function of tubular epithelial cells (instead of macrophages) to promote kidney repair in a mouse model of kidney ischemia–reperfusion (150). Recently, a strategy called “chimeric receptor for efferocytosis” was advanced to boost efferocytosis and facilitate the resolution of inflammation in mice (151). Tabas and Thorp revealed that MerTK shedding requires the metalloproteinase ADAM17 in a mouse model of endotoxemia (129). This type of MerTK cleavage during inflammation suppresses the biosynthesis of specialized pro-resolving mediators and boosts inflammation by inhibiting efferocytosis. Moreover, the authors developed a new MerTK-cleavage-resistant mouse model in which resistance to cleavage by metalloproteinases had been engineered, thereby retaining the efferocytosis capacity to improve resolution (152). Therefore, promoting MerTK cleavage by ADAM17 may present a new therapeutic avenue in the tumor microenvironment. The triggering receptor expressed on myeloid cells 2 (TREM2), a myeloid receptor in microglia, sustains microglial responses (153). Katzenelenbogen discovered novel Arg1^+^Trem2^+^regulatory myeloid cells through single-cell RNA sequencing, revealing an immunosuppressive role of TREM2 in cancer (154). Another study confirmed these findings and found that TREM2 deficiency and anti-TREM2 mAb treatment delayed the growth of transplanted tumors and enhanced anti-PD-1 immunotherapy in mice by remodeling the tumor macrophage landscape (155). However, it remains unclear whether this specific mechanism is related to efferocytosis. Given the wealth of recent studies on opportunities to treat diseases by targeting efferocytosis, we have summarized the therapeutic drugs below.

Tumor cells send “do not eat me” signal to macrophages through a high expression of CD47 to avoid being attacked and excluded by the innate immune system (156). Therapies for autoimmune or inflammatory diseases based on the modulation of efferocytosis have also been proposed. Magrolimab (Gilead Sciences, Foster City, CA, USA) is a CD47 antibody that is currently being tested in phase III clinical trials to treat acute myelocytic leukemia. Magrolimab relies on a laborious “pre-dose” regimen to reduce toxicity (e.g., anemia) to a certain extent. However, the blood toxicity caused by magrolimab is concerning. Simultaneously, the “antigen-sinking effect” caused by magrolimab binding to red blood cells indirectly affects its clinical efficacy (157, 158). In the search for methods to avoid blood toxicity, ALX148 (ALX Oncology, San Francisco, CA, USA) was identified as another approach. ALX148 is mainly used in phase I and phase II clinical studies, and its curative effect on B-cell non-Hodgkin’s lymphoma is currently in phase III clinical trials. The aim of ALX Oncology is to explore combined treatment approaches (159). AK117 (Akeso Biopharmaceuticals, Zhongshan, China), AO-176 (Arch Oncology, Brisbane, CA, USA), and HX009 (Waterstone Han X Bio, Beijing, China) are currently in phase I or phase II clinical trials (160, 161). Signal-regulated protein-α (SIRP-α) is a well-known ligand for CD47. Liu’s team engineered a specific nano-bioconjugate for macrophage-mediated atherosclerosis therapy. This nanotherapy showed a promising curative effect *in vitro* and *in vivo* with the combination of anti-SIRPα antibodies and antisense oligonucleotides of mTOR (162).

Sabatolimab (also called MBG453, Novartis Basel, Switzerland) targets Tim3/4 and is used to treat advanced malignancies, either alone or in combination with other antitumor medicines. Two other Tim3/4 targets, TSR-022 (Tersaro, Waltham, MA, USA) and LY3321367 (Eli Lilly, Indianapolis, IN, USA), have been used in the treatment of advanced solid tumors, and the clinical trials for these agents are in phase I and phase II (163).

Efforts are underway to develop drugs that target the TAM receptor family. Bemcentinib (BerGenBio ASA, Bergen, Norway), amuvatinib (Astex Pharmaceuticals, Cambridge, UK), cabozantini (Exelixis, Alameda, CA, USA), and TP-0903 (Sumitomo Dainippon Pharma Oncology, Cambridge, MA, USA) target the AXL receptor. Bemcentinib can stimulate antileukemic immunity and eradicate naïve and treatment-resistant leukemia. This blockade is effective as a PD-1 checkpoint blockade in PD-1-refractory leukemias (164). These drugs are mainly used in phase I and phase II clinical trials for lung cancer, solid tumors, and drug-resistant acute myeloid leukemia (165–167). Similarly, MerTK-mediated efferocytosis has been shown to promote metastatic tumor progression during postpartum mammary gland involution in mice (168). Anti-MerTK antibodies potentiate anti-tumor immunity (169) and decrease mammary tumor metastasis (168). Agents targeting on MerTK are in phase I and phase II clinical trials, including ONO-7475 (Ono Pharmaceuticals, Osaka, Japan), MRX-2843 (Meryx, Chapel Hill, NC, USA), and PF-07265807 (Pfizer, New York, NY, USA) (170–172). A lipid nanoparticle platform encapsulating siRNA for the phagocytic receptor MerTK (siMerTK) was found to selectively inhibit MerTK-mediated efferocytosis and exert therapeutic effects in both liver and peritoneal metastasis models of colorectal cancers. In the future, combining nanoparticles with immune checkpoint therapies (such as PD-1 blockade) may be a promising modality for metastatic colorectal cancer therapy (173).

Some metabolic pathway nodes can alter the immunological behavior of macrophages and consequently influence the homeostasis of the immune microenvironment. However, the anfractuosity and intricacy of metabolic networks hinder the development of metabolism-based therapies. Treatments targeting nucleotide metabolism were the earliest and most commonly developed (174). An increasing number of clinical trials have investigated non-nucleotide metabolic drugs targeting the electron transport chain (175, 176), asparagine synthetase (176), 3-hydroxy-3-methylglutaryl-CoA reductase (177), indoleamine 2,3-dioxygenase-1 (178), and mutated isocitrate dehydrogenase (IDH)-1. However, there are relatively few clinical trials involving dietary interventions, and the designs are relatively extensive (179). In general, except for nucleotide-metabolizing drugs, inhibitors of asparaginase synthetase, and IDH-1 inhibitors, other metabolism-targeting therapies are mostly in their infancy and have not yet achieved ideal therapeutic effects (180). The exploration of plausible targets within metabolic pathways that enhance efferocytosis and anti-inflammatory reactions requires considerable research.

**Figure 3:**
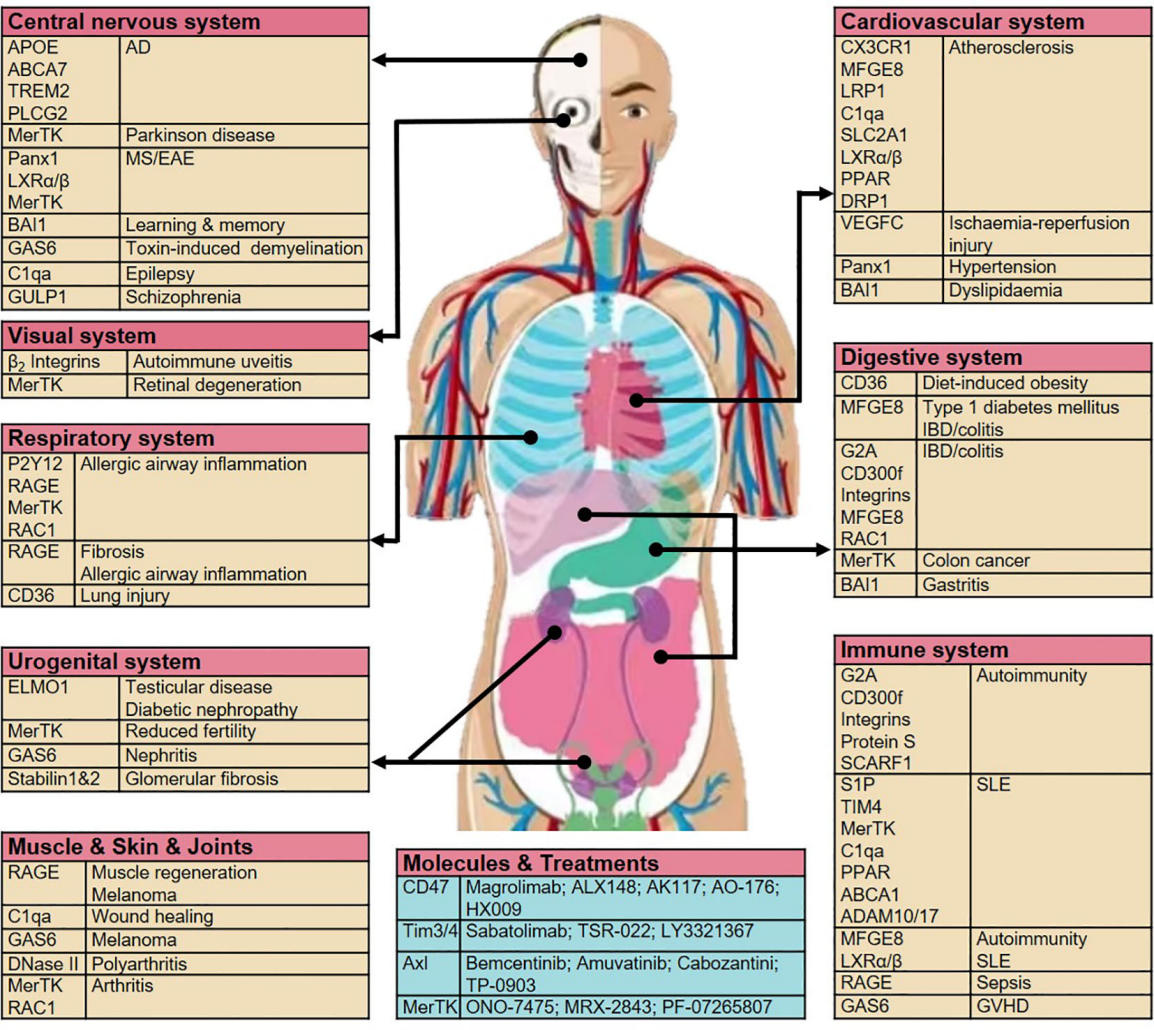
Aberrant efferocytosis by macrophages can result in a wide variety of diseases across various systems. Diseases associated with specific efferocytosis-related molecules and opportunities for targeting these molecules are shown. AD, Alzheimer’s disease; TREM2, triggering receptor expressed on myeloid cells 2; MS, multiple sclerosis; EAE, experimental autoimmune encephalomyelitis; BAI1, brain-specific angiogenesis inhibitor 1; GAS6, growth arrest-specific protein 6; C1qa, complement C1q subcomponent subunit A; GULP, PTB domain-containing engulfment adapter protein; LXR, liver X receptor; P2Y2, purinergic receptors; RAGE, receptor for advanced glycosylation end products; RAC1, Rac Family Small GTPase 1; ELMO1, engulfment and cell motility protein 1; CX3CR1, C-X3-C motif chemokine receptor; MFGE8, milk fat globule–EGF factor 8; LRP1, LDL receptor-related protein 1; SLC2A1, solute carrier family 2 member 1; PPAR, peroxisome proliferator-activated receptor; DRP1, dynamin-related protein 1; DOCK180, dedicator of cytokinesis protein 1; TIM4, T cell immunoglobulin mucin receptor 4; G2A, immunoglobulin G2a; IBD, inflammatory bowel disease; SCARF1, Scavenger receptor class F member 1; SLE, systemic lupus erythematosus; ABCA1, ATP-binding cassette transporter 1; VPS34, vacuolar protein sorting 34; ATG35,7,16, autophagy-related gene 35,7,16; GVHD, graft-*versus*-host disease.

Dangers and opportunities exist in the development of new targeted drugs; however, future selective targets for efferocytosis remain possible. Various system-related diseases associated with aberrant efferocytosis by macrophages and opportunities to target efferocytosis-related molecules are summarized in **Figure 3** and **Table 1**.

**Table 1:** Efferocytosis-targeting agents under clinical development.

Target	Agent	Alias(es)	Developer	Pathologies	Trial phase	Side effects	References
CD47	Hu5F9-G4	Magrolimab	Gilead Sciences	Solid tumor, AML	III	Anemia, thrombocytopenia	(159, 160)
	ALX148	N/A	ALX Oncology	Solid tumor, NHL	I/II/II	Not reported	(161)
	AK117	N/A	Akesobio Pharmaceuticals	Solid tumor, AML, MDS	I/II	Not reported	(163, 165)
	AO-176	N/A	Arch Oncology	Solid tumor	I/II	Not reported	
	HX009	N/A	Waterstone Han X Bio Pty Ltd	Solid tumor	I	Not reported	
Tim3/4	Sabatolimab	MBG453	Novartis	AML, MDS	I/II/III	Not reported	(165)
	TSR-022	N/A	Tersaro	Solid tumor	I/II	Not reported	
	LY3321367	N/A	Eli Lilly and Company	Solid tumor	I	Not reported	(166)
AXL	Bemcentinib	BGB324 R428	BerGenBio ASA	Solid tumor, AML, MDS	I/II	Not reported	(167, 170, 171)
	Amuvatinib	MP-470	Astex Pharmaceuticals	Solid tumor	I/II	Fatigue, alopecia, thrombocytopenia, leukopenia, anemia	
	Cabozantini	XL184 BMS-907351	Exelixis	Solid tumor, AML	I/II/III	Diarrhea, palmar plantar erythrodysesthesia syndrome, hypertension	
	Dubermatinib	TP-0903	Sumitomo Dainippon Pharma	Solid tumor, AML, CLL	I/II	Not reported	
MerTK	ONO-7475	N/A	Ono Pharmaceuticals	Solid tumor, AML	I/II	Not reported	(172, 174)
	MRX-2843	N/A	Meryx, Inc.	Solid tumor	I/II	Not reported	
	PF-07265807	N/A	Pfizer	Solid tumor	I	Not reported	(174)

AML, acute myeloid leukemia; MDS, myelodysplastic syndromes; CLL, chronic lymphocytic leukemia; NHL, non-Hodgkin lymphoma; N/A, not applicable.

## 5 Regulatory pathways and molecular mechanisms of efferocytosis

With the development of selective and potent biologics agents and compounds that can regulate efferocytosis selectively, developing some reliable methods to track cell death (and subsequent “corpse” removal) *in vivo* to reveal the mechanisms of efferocytosis will become crucial. Efferocytosis-related receptor–ligand interactions have been discovered; however, tracking efferocytosis *in vivo* is challenging. Detecting ACs *in vivo* is difficult because of their rapid removal and the lack of tools to track newly emerging ACs. Raymond et al. developed a genetically encoded fluorescent reporter program for *Drosophila* species to track emerging ACs and efferocytosis which can help uncover efferocytosis *in vivo* (181). Batoon et al. invented a novel inducible caspase-9 mouse model to achieve selective apoptosis and facilitate the examination of subsequent efferocytosis (182). Moreover, a genome-wide clustered regularly interspaced short palindromic repeat setup was created to screen for the regulators of efferocytosis by macrophages (183). In recent years, efferocytosis-related activation of metabolism that mediates macrophage reprogramming has received increasing attention in the field of immunology and metabolomics. We discuss below the metabolism of carbohydrates, cholesterol, fatty acids, amino acids, and nucleotides in macrophages during efferocytosis.

Carbohydrates are the most abundant macromolecules on earth and can be catabolized to provide energy (ATP) or anabolized to maintain vital activities. Carbohydrates comprise three major groups: (i) monosaccharides and disaccharides (e.g., glucose), (ii) complex carbohydrates (e.g., glycogen), and (iii) glycoconjugates, (glycoproteins, glycolipids) (184). Glycolysis, OXPHOS, and the pentose phosphate pathway (PPP) are critical for macrophage reprogramming. Glycolysis is known to be related to the proinflammatory phenotype of tumor-associated macrophages (185); however, this view has been challenged since a recent study revealed that glycolysis is increased in anti-inflammatory efferocytic macrophages (42). Glucose in macrophages undergoing efferocytosis arises mainly from the extracellular matrix transported by GLUT1 and degraded by apoptotic vesicles. Glycolysis of glucose produces pyruvate, which is converted to lactic acid or transferred to the inner mitochondrial membrane to enter the tricarboxylic acid (TCA) cycle. Lactate in macrophages helps to inhibit inflammation (42) and promotes sustained efferocytosis by macrophages through interactions with MerTK and LRP1 (186). Lactate combines with G protein-coupled receptor-132 (187, 188) to activate downstream AMPK, which promotes mitochondrial homeostasis (189) and the proliferation of pro-resolving macrophages (190). In addition to being involved in glycolysis, intracellular glucose is involved in the PPP by transforming into glucose 6-phosphate (191). A previous study suggested that efferocytosis and the PPP are mutually inhibitory (192). Moreover, a recent study demonstrated that reduced nicotinamide adenine dinucleotide phosphate from the PPP loop contributes to efferocytosis by macrophages under prolonged (chronic) physiological hypoxia (193).

Cholesterol from degraded vesicles can support efferocytosis, promote the repair of inflammation *via* LXRs, and act on MerTK/LRP1 to favor further recognition and phagocytosis (194). The ABCA1/ABCG1-mediated cholesterol efflux balances the amount of cholesterol in macrophages (195). Another study found that Niemann–Pick-type C1-related cholesterol extraction is required for the ongoing phagocytic activity of macrophages and may be a therapeutic target in the future (196). Statins lower intracellular cholesterol levels to prevent uncontrolled inflammation by regulating efferocytosis by macrophages (43). ω-3 free fatty acids from ACs produce specialized pro-resolving mediators (SPMs) by 12/15-lipoxygenase. SPMs can promote efferocytosis and resolution of inflammation (197, 198). Macrophages convert docosahexaenoic acid to maresin conjugates in tissue regeneration (MCTRs) with 12-lipoxygenase. MCTRs contribute to continuous efferocytosis by macrophages through the Rac1-mediated activation of glycolysis (199).

Glutamine participates in the TCA cycle *via* glutaminase-1 and promotes macrophage proliferation (200). Polyamine accumulation in the macrophage cytoplasm inhibits the secretion of interleukin (IL)-1β and IL-6 (201). Arginine from ACs is transformed to putrescine to activate RAC1 to promote continual efferocytosis (45), and this arginine metabolism can be regulated by 3,3′-diindolylmethane (202). Indoleamine-2,3-dioxegenase1 promotes the transformation of tryptophan to kynurenine, and the latter enhances the expression of IL-10 and transforming growth factor-β (203, 204). Additionally, AC-derived methionine transforms to S-adenosylmethionine, and the latter contributes to enhancing the expression of transforming growth factor-β *via* DNA methyltransferase-3A (205).

**Figure 4:**
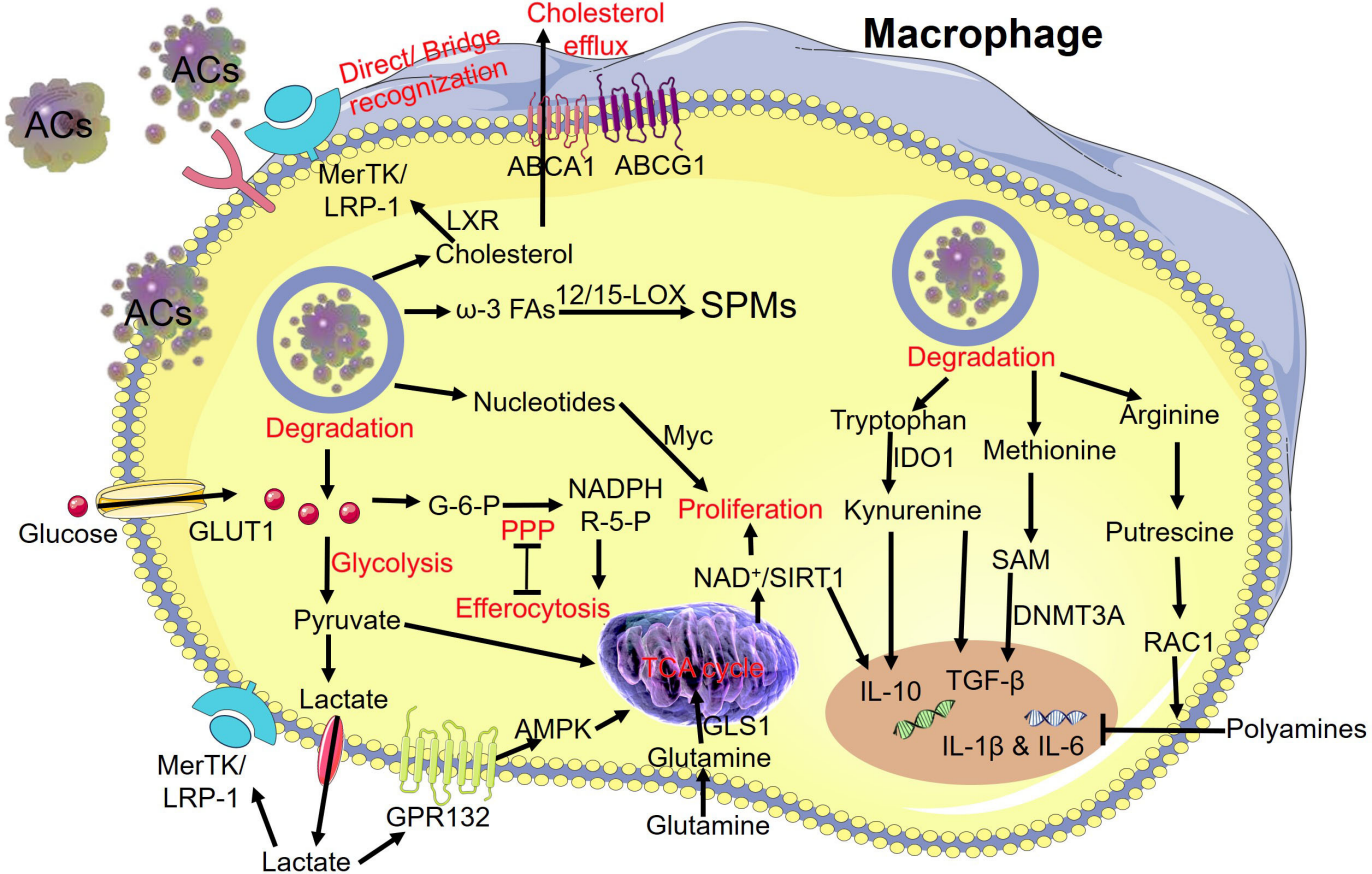
Degradation of metabolites from “apoptotic debris” and subsequent reprogramming of macrophages during efferocytosis. The release of carbohydrates, lipids, amino acids, and nucleic acids from the AC cargo modulates the metabolic reprogramming of macrophages. MerTK, MER proto-oncogene tyrosine kinase; LRP-1, low-density lipoprotein receptor-related protein 1; LXR, the nuclear hormone receptor; ABCA1, ATP-binding cassette transporter A1; ABCG1, ATP-binding cassette transporter G1; ω-3 FAs, ω-3 fatty acids; 12/15-LOX, 12/15-lipoxygenase; SPMs, specialized pro-resolving mediators; GLUT1, glucose transporter; G-6-P, glucose 6-phosphate; NADPH, nicotinamide adenine dinucleotide phosphate; R-5-P, ribose-5-phosphate; PPP, pentose phosphate pathway; AMPK, adenosine monophosphate activated protein kinase; GPR132, G-protein coupled receptors; TCA cycle, tricarboxylic acid cycle; GLS1, glutaminase 1; SIRT1, sirtuin 1; IDO1, indoleamine 2,3 dioxygenase 1; IL-10, interleukin 10; TGF-β, transforming growth factor-β; SAM, S-adenosylmethionine; DNMT3A, DNA-methyltransferase-3A.

In summary, metabolites and signaling molecules from ACs activate a complex regulatory network during efferocytosis and further enhance the immunological behavior of macrophages (**Figure 4**).

## 6 Discussion and future directions

Three key aspects of efferocytosis and metabolic mechanism during the digestion stage are presented in **Figures 2**, **4**. **Figure 3** displays the diseases associated with aberrant macrophage efferocytosis in different systems and highlights the opportunities for targeting efferocytosis-related molecules. However, our understanding of efferocytosis remains incomplete. Over the next decade, at least three key questions remain to be answered, which may drive advances in efferocytosis research.

First, the factors determining macrophage turnover and lifespan during efferocytosis are incompletely understood. These efferocytosis-related phagocytes activate apoptosis and necrosis in neighboring cells, in addition to extensive efferocytosis (206). However, the mechanisms and features of these biological processes require further study. Second, the functions of the various molecules involved in the reprogramming of macrophages during efferocytosis remain unknown—for example, the role of molecules in the glycolytic pathway in efferocytosis and the function of macrophages, together with their clinical application, merit further research. Third, the methods for selectively controlling efferocytosis are not yet known, including whether the types of cell death can be adjusted and whether the molecules associated with efferocytosis can be targeted. Selective targeting of SPMs may be helpful.

Efferocytosis can be selectively controlled in specific contexts using several methods, including pharmacokinetics, optimized biodistribution, and drug delivery. Addressing these strategies and mechanisms will serve to contribute to the knowledge of efferocytosis and could also yield therapeutic benefits for systemic diseases. Insights into the increasingly diverse areas of biology related to efferocytosis will continue to be renewed.

## Author contributions

YRS: Writing – original draft, Funding acquisition. WTH: Writing – review & editing. SMC: Writing – review & editing. XYZ: Writing – review & editing, Funding acquisition, Supervision.

## References

[1] deCathelineau AM Henson PM. The final step in programmed cell death: phagocytes carry apoptotic cells to the grave. Essays Biochem. (2003) 39:105–17. doi: 10.1042/bse0390105 14585077

[2] Canton J Khezri R Glogauer M Grinstein S. Contrasting phagosome ph regulation and maturation in human M1 and M2 macrophages. Mol Biol Cell. (2014) 25:3330–41. doi: 10.1091/mbc.E14-05-0967 PMC4214780 25165138

[3] Fadok VA Xue D Henson P. If phosphatidylserine is the death knell, a new phosphatidylserine-specific receptor is the bellringer. Cell Death differentiation. (2001) 8:582–7. doi: 10.1038/sj.cdd.4400856 11536008

[4] Ravichandran KS. "Recruitment signals" from apoptotic cells: invitation to a quiet meal. Cell. (2003) 113:817–20. doi: 10.1016/s0092-8674(03)00471-9 12837239

[5] Bertheloot D Latz E Franklin BS. Necroptosis, pyroptosis and apoptosis: an intricate game of cell death. Cell Mol Immunol. (2021) 18:1106–21. doi: 10.1038/s41423-020-00630-3 PMC8008022 33785842

[6] Andrejeva G Rathmell JC. Similarities and distinctions of cancer and immune metabolism in inflammation and tumors. Cell Metab. (2017) 26:49–70. doi: 10.1016/j.cmet.2017.06.004 28683294 PMC5555084

[7] Metschnikoff E. Lecture on phagocytosis and immunity. Br Med J. (1891) 1:213–7. doi: 10.1136/bmj.1.1570.213 PMC2197023 20753232

[8] Rabinovitch M. Professional and non-professional phagocytes: an introduction. Trends Cell Biol. (1995) 5:85–7. doi: 10.1016/s0962-8924(00)88955-2 14732160

[9] AG N Quintana JA García-Silva S Mazariegos M González de la Aleja A Nicolás-Ávila JA. Phagocytosis imprints heterogeneity in tissue-resident macrophages. J Exp Med. (2017) 214:1281–96. doi: 10.1084/jem.20161375 PMC5413334 28432199

[10] Lockshin RA Williams CM. Programmed cell death–I. Cytology of degeneration in the intersegmental muscles of the pernyi silkmoth. J Insect Physiol. (1965) 11:123–33. doi: 10.1016/0022-1910(65)90099-5 14287218

[11] De Maria R Lenti L Malisan F d'Agostino F Tomassini B Zeuner A. Requirement for gd3 ganglioside in cd95- and ceramide-induced apoptosis. Sci (New York NY). (1997) 277:1652–5. doi: 10.1126/science.277.5332.1652 9287216

[12] Deter RL De Duve C. Influence of glucagon, an inducer of cellular autophagy, on some physical properties of rat liver lysosomes. J Cell Biol. (1967) 33:437–49. doi: 10.1083/jcb.33.2.437 PMC2108350 4292315

[13] Fernandes-Alnemri T Wu J Yu JW Datta P Miller B Jankowski W. The pyroptosome: A supramolecular assembly of asc dimers mediating inflammatory cell death *via* caspase-1 activation. Cell Death differentiation. (2007) 14:1590–604. doi: 10.1038/sj.cdd.4402194 PMC3345951 17599095

[14] Tang D Chen X Kroemer G. Cuproptosis: A copper-triggered modality of mitochondrial cell death. Cell Res. (2022) 32:417–8. doi: 10.1038/s41422-022-00653-7 PMC9061796 35354936

[15] Dixon SJ Lemberg KM Lamprecht MR Skouta R Zaitsev EM Gleason CE. Ferroptosis: an iron-dependent form of nonapoptotic cell death. Cell. (2012) 149:1060–72. doi: 10.1016/j.cell.2012.03.042 PMC3367386 22632970

[16] Teng X Degterev A Jagtap P Xing X Choi S Denu R. Structure-activity relationship study of novel necroptosis inhibitors. Bioorganic medicinal Chem Lett. (2005) 15:5039–44. doi: 10.1016/j.bmcl.2005.07.077 16153840

[17] Bousselin M. Observations on necrosis. London Med J. (1786) 7:263–79. PMC5545397 29139835

[18] Shiratori-Aso S Nakazawa D Kudo T Kanda M Ueda Y Watanabe-Kusunoki K. Cd47 blockade ameliorates autoimmune vasculitis *via* efferocytosis of neutrophil extracellular traps. JCI Insight. (2023) 8. doi: 10.1172/jci.insight.167486 PMC10445685 37368493

[19] Sheng YR Hu WT Shen HH Wei CY Liu YK Ma XQ. An imbalance of the il-33/st2-axl-efferocytosis axis induces pregnancy loss through metabolic reprogramming of decidual macrophages. Cell Mol Life sciences: CMLS. (2022) 79:173. doi: 10.1007/s00018-022-04197-2 35244789 PMC11073329

[20] Dini L Autuori F Lentini A Oliverio S Piacentini M. The clearance of apoptotic cells in the liver is mediated by the asialoglycoprotein receptor. FEBS Lett. (1992) 296:174–8. doi: 10.1016/0014-5793(92)80373-O 1370803

[21] Sadik CD Bachmann M Pfeilschifter J Mühl H. Activation of interferon regulatory factor-3 *via* toll-like receptor 3 and immunomodulatory functions detected in A549 lung epithelial cells exposed to misplaced U1-snrna. Nucleic Acids Res. (2009) 37:5041–56. doi: 10.1093/nar/gkp525 PMC2731906 19541850

[22] Vachon E Martin R Plumb J Kwok V Vandivier RW Glogauer M. Cd44 is a phagocytic receptor. Blood. (2006) 107:4149–58. doi: 10.1182/blood-2005-09-3808 16455948

[23] Kobayashi N Karisola P Peña-Cruz V Dorfman DM Jinushi M Umetsu SE. Tim-1 and tim-4 glycoproteins bind phosphatidylserine and mediate uptake of apoptotic cells. Immunity. (2007) 27:927–40. doi: 10.1016/j.immuni.2007.11.011 PMC2757006 18082433

[24] Nakayama M Akiba H Takeda K Kojima Y Hashiguchi M Azuma M. Tim-3 mediates phagocytosis of apoptotic cells and cross-presentation. Blood. (2009) 113:3821–30. doi: 10.1182/blood-2008-10-185884 19224762

[25] Cheong HS Lee SO Choi CB Sung YK Shin HD Bae SC. Mertk polymorphisms associated with risk of haematological disorders among Korean sle patients. Rheumatol (Oxford England). (2007) 46:209–14. doi: 10.1093/rheumatology/kel182 16837475

[26] Lemke G Rothlin CV. Immunobiology of the tam receptors. Nat Rev Immunol. (2008) 8:327–36. doi: 10.1038/nri2303 PMC2856445 18421305

[27] Park SY Kang KB Thapa N Kim SY Lee SJ Kim IS. Requirement of adaptor protein gulp during stabilin-2-mediated cell corpse engulfment. J Biol Chem. (2008) 283:10593–600. doi: 10.1074/jbc.M709105200 18230608

[28] Baruah P Dumitriu IE Peri G Russo V Mantovani A Manfredi AA. The tissue pentraxin ptx3 limits C1q-mediated complement activation and phagocytosis of apoptotic cells by dendritic cells. J leukocyte Biol. (2006) 80:87–95. doi: 10.1189/jlb.0805445 16617159

[29] Gronski MA Kinchen JM Juncadella IJ Franc NC Ravichandran KS. An essential role for calcium flux in phagocytes for apoptotic cell engulfment and the anti-inflammatory response. Cell Death differentiation. (2009) 16:1323–31. doi: 10.1038/cdd.2009.55 PMC2856475 19461656

[30] AG N Bensinger SJ Hong C Beceiro S Bradley MN Zelcer N. Apoptotic cells promote their own clearance and immune tolerance through activation of the nuclear receptor lxr. Immunity. (2009) 31:245–58. doi: 10.1016/j.immuni.2009.06.018 PMC2791787 19646905

[31] Chen D Xiao H Zhang K Wang B Gao Z Jian Y. Retromer is required for apoptotic cell clearance by phagocytic receptor recycling. Sci (New York NY). (2010) 327:1261–4. doi: 10.1126/science.1184840 20133524

[32] Elliott MR Zheng S Park D Woodson RI Reardon MA Juncadella IJ. Unexpected requirement for elmo1 in clearance of apoptotic germ cells in vivo. Nature. (2010) 467:333–7. doi: 10.1038/nature09356 PMC3773546 20844538

[33] Park D Han CZ Elliott MR Kinchen JM Trampont PC Das S. Continued clearance of apoptotic cells critically depends on the phagocyte ucp2 protein. Nature. (2011) 477:220–4. doi: 10.1038/nature10340 PMC3513690 21857682

[34] Hsu CL Lin W Seshasayee D Chen YH Ding X Lin Z. Equilibrative nucleoside transporter 3 deficiency perturbs lysosome function and macrophage homeostasis. Sci (New York NY). (2012) 335:89–92. doi: 10.1126/science.1213682 22174130

[35] Neukomm LJ Frei AP Cabello J Kinchen JM Zaidel-Bar R Ma Z. Loss of the rhogap srgp-1 promotes the clearance of dead and injured cells in caenorhabditis elegans. Nat Cell Biol. (2011) 13:79–86. doi: 10.1038/ncb2138 21170032 PMC3808961

[36] Prabagar MG Do Y Ryu S Park JY Choi HJ Choi WS. Sign-R1, a C-type lectin, enhances apoptotic cell clearance through the complement deposition pathway by interacting with C1q in the spleen. Cell Death differentiation. (2013) 20:535–45. doi: 10.1038/cdd.2012.160 PMC3595488 23238564

[37] Ramirez-Ortiz ZG Pendergraft WF 3rd Prasad A Byrne MH Iram T Blanchette CJ. The scavenger receptor scarf1 mediates the clearance of apoptotic cells and prevents autoimmunity. Nat Immunol. (2013) 14:917–26. doi: 10.1038/ni.2670 PMC3752698 23892722

[38] Yoon KW Byun S Kwon E Hwang SY Chu K Hiraki M. Control of signaling-mediated clearance of apoptotic cells by the tumor suppressor P53. Sci (New York NY). (2015) 349:1261669. doi: 10.1126/science.1261669 PMC5215039 26228159

[39] Luo B Gan W Liu Z Shen Z Wang J Shi R. Erythropoeitin signaling in macrophages promotes dying cell clearance and immune tolerance. Immunity. (2016) 44:287–302. doi: 10.1016/j.immuni.2016.01.002 26872696

[40] Kojima Y Volkmer JP McKenna K Civelek M Lusis AJ Miller CL. Cd47-blocking antibodies restore phagocytosis and prevent atherosclerosis. Nature. (2016) 536:86–90. doi: 10.1038/nature18935 27437576 PMC4980260

[41] Han CZ Juncadella IJ Kinchen JM Buckley MW Klibanov AL Dryden K. Macrophages redirect phagocytosis by non-professional phagocytes and influence inflammation. Nature. (2016) 539:570–4. doi: 10.1038/nature20141 PMC5799085 27820945

[42] Morioka S Perry JSA Raymond MH Medina CB Zhu Y Zhao L. Efferocytosis induces a novel slc program to promote glucose uptake and lactate release. Nature. (2018) 563:714–8. doi: 10.1038/s41586-018-0735-5 PMC6331005 30464343

[43] Tajbakhsh A Gheibihayat SM Askari H Savardashtaki A Pirro M Johnston TP. Statin-regulated phagocytosis and efferocytosis in physiological and pathological conditions. Pharmacol Ther. (2022) 238:108282. doi: 10.1016/j.pharmthera.2022.108282 36130624

[44] Hosseini Z Marinello M Decker C Sansbury BE Sadhu S Gerlach BD. Resolvin D1 enhances necroptotic cell clearance through promoting macrophage fatty acid oxidation and oxidative phosphorylation. Arteriosclerosis thrombosis Vasc Biol. (2021) 41:1062–75. doi: 10.1161/atvbaha.120.315758 PMC8174560 33472399

[45] Yurdagul A Jr. Subramanian M Wang X Crown SB Ilkayeva OR Darville L. Macrophage metabolism of apoptotic cell-derived arginine promotes continual efferocytosis and resolution of injury. Cell Metab. (2020) 31:518–33.e10. doi: 10.1016/j.cmet.2020.01.001 32004476 PMC7173557

[46] Wu MY Liu L Wang EJ Xiao HT Cai CZ Wang J. Pi3kc3 complex subunit nrbf2 is required for apoptotic cell clearance to restrict intestinal inflammation. Autophagy. (2021) 17:1096–111. doi: 10.1080/15548627.2020.1741332 PMC8143223 32160108

[47] Tao J Zhang J Ling Y McCall CE Liu TF. Mitochondrial sirtuin 4 resolves immune tolerance in monocytes by rebalancing glycolysis and glucose oxidation homeostasis. Front Immunol. (2018) 9:419. doi: 10.3389/fimmu.2018.00419 29593712 PMC5854658

[48] Xu M Zhou C Weng J Chen Z Zhou Q Gao J. Tumor associated macrophages-derived exosomes facilitate hepatocellular carcinoma Malignance by transferring lncmmpa to tumor cells and activating glycolysis pathway. J Exp Clin Cancer research: CR. (2022) 41:253. doi: 10.1186/s13046-022-02458-3 35986343 PMC9389814

[49] Gonzalez MA Lu DR Yousefi M Kroll A Lo CH Briseño CG. Phagocytosis increases an oxidative metabolic and immune suppressive signature in tumor macrophages. J Exp Med. (2023) 220. doi: 10.1084/jem.20221472 PMC10067971 36995340

[50] Yu C Niu X Du Y Chen Y Liu X Xu L. Il-17a promotes fatty acid uptake through the il-17a/il-17ra/P-stat3/fabp4 axis to fuel ovarian cancer growth in an adipocyte-rich microenvironment. Cancer immunology immunotherapy: CII. (2020) 69:115–26. doi: 10.1007/s00262-019-02445-2 PMC11027837 31802182

[51] Batista-Gonzalez A Vidal R Criollo A Carreño LJ. New insights on the role of lipid metabolism in the metabolic reprogramming of macrophages. Front Immunol. (2019) 10:2993. doi: 10.3389/fimmu.2019.02993 31998297 PMC6966486

[52] Vitale I Manic G Coussens LM Kroemer G Galluzzi L. Macrophages and metabolism in the tumor microenvironment. Cell Metab. (2019) 30:36–50. doi: 10.1016/j.cmet.2019.06.001 31269428

[53] Geeraerts X Fernández-Garcia J Hartmann FJ de Goede KE Martens L Elkrim Y. Macrophages are metabolically heterogeneous within the tumor microenvironment. Cell Rep. (2021) 37:110171. doi: 10.1016/j.celrep.2021.110171 34965415

[54] Su P Wang Q Bi E Ma X Liu L Yang M. Enhanced lipid accumulation and metabolism are required for the differentiation and activation of tumor-associated macrophages. Cancer Res. (2020) 80:1438–50. doi: 10.1158/0008-5472.Can-19-2994 PMC7127942 32015091

[55] Leone RD Zhao L Englert JM Sun IM Oh MH Sun IH. Glutamine blockade induces divergent metabolic programs to overcome tumor immune evasion. Sci (New York NY). (2019) 366:1013–21. doi: 10.1126/science.aav2588 PMC7023461 31699883

[56] Jia Q Wang A Yuan Y Zhu B Long H. Heterogeneity of the tumor immune microenvironment and its clinical relevance. Exp Hematol Oncol. (2022) 11:24. doi: 10.1186/s40164-022-00277-y 35461288 PMC9034473

[57] Meagher LC Savill JS Baker A Fuller RW Haslett C. Phagocytosis of apoptotic neutrophils does not induce macrophage release of thromboxane B2. J leukocyte Biol. (1992) 52:269–73. doi: 10.1002/jlb.52.3.269 1522386

[58] Yu L Zhu G Zhang Z Yu Y Zeng L Xu Z. Apoptotic bodies: bioactive treasure left behind by the dying cells with robust diagnostic and therapeutic application potentials. J nanobiotechnology. (2023) 21:218. doi: 10.1186/s12951-023-01969-1 37434199 PMC10337089

[59] Zhang S Weinberg S DeBerge M Gainullina A Schipma M Kinchen JM. Efferocytosis fuels requirements of fatty acid oxidation and the electron transport chain to polarize macrophages for tissue repair. Cell Metab. (2019) 29:443–56.e5. doi: 10.1016/j.cmet.2018.12.004 30595481 PMC6471613

[60] Kamei R Okabe S. *In vivo* imaging of the phagocytic dynamics underlying efficient clearance of adult-born hippocampal granule cells by ramified microglia. Glia. (2023) 71:2005–23. doi: 10.1002/glia.24379 37102766

[61] Cui C Wang F Zheng Y Wei H Peng J. From birth to death: the hardworking life of paneth cell in the small intestine. Front Immunol. (2023) 14:1122258. doi: 10.3389/fimmu.2023.1122258 36969191 PMC10036411

[62] Grootveld AK Kyaw W Panova V Lau AWY Ashwin E Seuzaret G. Apoptotic cell fragments locally activate tingible body macrophages in the germinal center. Cell. (2023) 186:1144–61.e18. doi: 10.1016/j.cell.2023.02.004 36868219 PMC7614509

[63] Ardavín C Alvarez-Ladrón N Ferriz M Gutiérrez-González A Vega-Pérez A. Mouse tissue-resident peritoneal macrophages in homeostasis, repair, infection, and tumor metastasis. Advanced Sci (Weinheim Baden-Wurttemberg Germany). (2023) 10:e2206617. doi: 10.1002/advs.202206617 PMC10104642 36658699

[64] Gu Y Zhou Y Ju S Liu X Zhang Z Guo J. Multi-omics profiling visualizes dynamics of cardiac development and functions. Cell Rep. (2022) 41:111891. doi: 10.1016/j.celrep.2022.111891 36577384

[65] Abrahams VM Kim YM Straszewski SL Romero R Mor G. Macrophages and apoptotic cell clearance during pregnancy. Am J Reprod Immunol (New York NY: 1989). (2004) 51:275–82. doi: 10.1111/j.1600-0897.2004.00156.x 15212680

[66] Zuttion M Parimon T Yao C Stripp BR Wang Y Soto CM. Interstitial macrophages mediate efferocytosis of alveolar epithelium during influenza infection. Am J Respir Cell Mol Biol. (2024). doi: 10.1165/rcmb.2023-0217MA PMC10914771 38207122

[67] Schloesser D Lindenthal L Sauer J Chung KJ Chavakis T Griesser E. Senescent cells suppress macrophage-mediated corpse removal *via* upregulation of the cd47-qpct/L axis. J Cell Biol. (2023) 222. doi: 10.1083/jcb.202207097 PMC9723804 36459066

[68] Hu H Cheng X Li F Guan Z Xu J Wu D. Defective efferocytosis by aged macrophages promotes sting signaling mediated inflammatory liver injury. Cell Death Discovery. (2023) 9:236. doi: 10.1038/s41420-023-01497-9 37422464 PMC10329662

[69] Monks J Smith-Steinhart C Kruk ER Fadok VA Henson PM. Epithelial cells remove apoptotic epithelial cells during post-lactation involution of the mouse mammary gland. Biol Reprod. (2008) 78:586–94. doi: 10.1095/biolreprod.107.065045 18057312

[70] Quarato ER Salama NA Li AJ Smith CO Zhang J Kawano Y. Efferocytosis by bone marrow mesenchymal stromal cells disrupts osteoblastic differentiation *via* mitochondrial remodeling. Cell Death Dis. (2023) 14:428. doi: 10.1038/s41419-023-05931-9 37452070 PMC10349065

[71] Wu D Zhang K Khan FA Pandupuspitasari NS Liang W Huang C. Smtnl2 regulates apoptotic germ cell clearance and lactate metabolism in mouse sertoli cells. Mol Cell Endocrinol. (2022) 551:111664. doi: 10.1016/j.mce.2022.111664 35551947

[72] Rieu Q Bougoüin A Zagar Y Chatagnon J Hamieh A Enderlin J. Pleiotropic roles of scavenger receptors in circadian retinal phagocytosis: A new function for lysosomal sr-B2/limp-2 at the rpe cell surface. Int J Mol Sci. (2022) 23. doi: 10.3390/ijms23073445 PMC8998831 35408805

[73] Esmann L Idel C Sarkar A Hellberg L Behnen M Möller S. Phagocytosis of apoptotic cells by neutrophil granulocytes: diminished proinflammatory neutrophil functions in the presence of apoptotic cells. J Immunol (Baltimore Md: 1950). (2010) 184:391–400. doi: 10.4049/jimmunol.0900564 19949068

[74] Hellberg L Fuchs S Gericke C Sarkar A Behnen M Solbach W. Proinflammatory stimuli enhance phagocytosis of apoptotic cells by neutrophil granulocytes. TheScientificWorldJournal. (2011) 11:2230–6. doi: 10.1100/2011/413271 PMC3217599 22125470

[75] Schimek V Strasser K Beer A Göber S Walterskirchen N Brostjan C. Tumour cell apoptosis modulates the colorectal cancer immune microenvironment *via* interleukin-8-dependent neutrophil recruitment. Cell Death Dis. (2022) 13:113. doi: 10.1038/s41419-022-04585-3 35121727 PMC8816934

[76] Szondy Z Garabuczi E Joós G Tsay GJ Sarang Z. Impaired clearance of apoptotic cells in chronic inflammatory diseases: therapeutic implications. Front Immunol. (2014) 5:354. doi: 10.3389/fimmu.2014.00354 25136342 PMC4117929

[77] Tajbakhsh A Gheibi Hayat SM Butler AE Sahebkar A. Effect of soluble cleavage products of important receptors/ligands on efferocytosis: their role in inflammatory, autoimmune and cardiovascular disease. Ageing Res Rev. (2019) 50:43–57. doi: 10.1016/j.arr.2019.01.007 30639340

[78] Cummings CT Deryckere D Earp HS Graham DK. Molecular pathways: mertk signaling in cancer. Clin Cancer Res. (2013) 19:5275–80. doi: 10.1158/1078-0432.Ccr-12-1451 PMC10039640 23833304

[79] Romero-Molina C Garretti F Andrews SJ Marcora E Goate AM. Microglial efferocytosis: diving into the alzheimer's disease gene pool. Neuron. (2022) 110:3513–33. doi: 10.1016/j.neuron.2022.10.015 PMC13175419 36327897

[80] Adamson SE Leitinger N. The role of pannexin1 in the induction and resolution of inflammation. FEBS Lett. (2014) 588:1416–22. doi: 10.1016/j.febslet.2014.03.009 PMC4060616 24642372

[81] Lutz SE González-Fernández E Ventura JC Pérez-Samartín A Tarassishin L Negoro H. Contribution of pannexin1 to experimental autoimmune encephalomyelitis. PloS One. (2013) 8:e66657. doi: 10.1371/journal.pone.0066657 23885286 PMC3688586

[82] Kidani Y Bensinger SJ. Liver X receptor and peroxisome proliferator-activated receptor as integrators of lipid homeostasis and immunity. Immunol Rev. (2012) 249:72–83. doi: 10.1111/j.1600-065X.2012.01153.x 22889216 PMC4007066

[83] Wium M Paccez JD Zerbini LF. The dual role of tam receptors in autoimmune diseases and cancer: an overview. Cells. (2018) 7. doi: 10.3390/cells7100166 PMC6210017 30322068

[84] Obot P Subah G Schonwald A Pan J Velíšek L Velíšková J. Astrocyte and neuronal panx1 support long-term reference memory in mice. ASN Neuro. (2023) 15:17590914231184712. doi: 10.1177/17590914231184712 37365910 PMC10326369

[85] Cork SM Van Meir EG. Emerging roles for the bai1 protein family in the regulation of phagocytosis, synaptogenesis, neurovasculature, and tumor development. J Mol Med (Berlin Germany). (2011) 89:743–52. doi: 10.1007/s00109-011-0759-x PMC3152611 21509575

[86] Duman JG Tu YK Tolias KF. Emerging roles of bai adhesion-gpcrs in synapse development and plasticity. Neural plasticity. (2016) 2016:8301737. doi: 10.1155/2016/8301737 26881134 PMC4736325

[87] Chu Y Jin X Parada I Pesic A Stevens B Barres B. Enhanced synaptic connectivity and epilepsy in C1q knockout mice. Proc Natl Acad Sci United States America. (2010) 107:7975–80. doi: 10.1073/pnas.0913449107 PMC2867906 20375278

[88] Chen X Sun C Chen Q O'Neill FA Walsh D Fanous AH. Apoptotic engulfment pathway and schizophrenia. PloS One. (2009) 4:e6875. doi: 10.1371/journal.pone.0006875 19721717 PMC2731162

[89] Schittenhelm L Hilkens CM Morrison VL. B(2) integrins as regulators of dendritic cell, monocyte, and macrophage function. Front Immunol. (2017) 8:1866. doi: 10.3389/fimmu.2017.01866 29326724 PMC5742326

[90] Burstyn-Cohen T Lew ED Través PG Burrola PG Hash JC Lemke G. Genetic dissection of tam receptor-ligand interaction in retinal pigment epithelial cell phagocytosis. Neuron. (2012) 76:1123–32. doi: 10.1016/j.neuron.2012.10.015 PMC3530147 23259948

[91] Khaket TP Kang SC Mukherjee TK. The potential of receptor for advanced glycation end products (Rage) as a therapeutic target for lung associated diseases. Curr Drug Targets. (2019) 20:679–89. doi: 10.2174/1389450120666181120102159 30457049

[92] Cattaneo M. The platelet P2 receptors in inflammation. Hamostaseologie. (2015) 35:262–6. doi: 10.5482/hamo-14-09-0044 25579761

[93] Henson PM Bratton DL. Allergy: airway epithelial rac1 suppresses allergic inflammation. Curr biology: CB. (2013) 23:R104–6. doi: 10.1016/j.cub.2012.12.008 23391381

[94] Suresh K Servinsky L Reyes J Undem C Zaldumbide J Rentsendorj O. Cd36 mediates H2o2-induced calcium influx in lung microvascular endothelial cells. Am J Physiol Lung Cell Mol Physiol. (2017) 312:L143–l53. doi: 10.1152/ajplung.00361.2016 PMC5283925 27913425

[95] Sainaghi PP Bellan M Nerviani A. Role of the gas6/tam system as a disease marker and potential drug target. Dis Markers. (2021) 2021:2854925. doi: 10.1155/2021/2854925 33532004 PMC7834835

[96] Li BZ Zhang HY Pan HF Ye DQ. Identification of mfg-E8 as a novel therapeutic target for diseases. Expert Opin Ther Targets. (2013) 17:1275–85. doi: 10.1517/14728222.2013.829455 23972256

[97] Ruotsalainen SE Surakka I Mars N Karjalainen J Kurki M Kanai M. Inframe insertion and splice site variants in mfge8 associate with protection against coronary atherosclerosis. Commun Biol. (2022) 5:802. doi: 10.1038/s42003-022-03552-0 35978133 PMC9385630

[98] Márquez AB van der Vorst EPC Maas SL. Key chemokine pathways in atherosclerosis and their therapeutic potential. J Clin Med. (2021) 10. doi: 10.3390/jcm10173825 PMC8432216 34501271

[99] Boucher P Herz J. Signaling through lrp1: protection from atherosclerosis and beyond. Biochem Pharmacol. (2011) 81:1–5. doi: 10.1016/j.bcp.2010.09.018 20920479 PMC2991482

[100] Bhatia VK Yun S Leung V Grimsditch DC Benson GM Botto MB. Complement C1q reduces early atherosclerosis in low-density lipoprotein receptor-deficient mice. Am J Pathol. (2007) 170:416–26. doi: 10.2353/ajpath.2007.060406 PMC1762701 17200212

[101] Haskard DO Boyle JJ Mason JC. The role of complement in atherosclerosis. Curr Opin lipidology. (2008) 19:478–82. doi: 10.1097/MOL.0b013e32830f4a06 18769228

[102] Marino M Del Bo C Tucci M Venturi S Mantegazza G Taverniti V. A mix of chlorogenic and caffeic acid reduces C/ebpß and ppar-Γ1 levels and counteracts lipid accumulation in macrophages. Eur J Nutr. (2022) 61:1003–14. doi: 10.1007/s00394-021-02714-w 34698900

[103] Rogers MA Maldonado N Hutcheson JD Goettsch C Goto S Yamada I. Dynamin-related protein 1 inhibition attenuates cardiovascular calcification in the presence of oxidative stress. Circ Res. (2017) 121:220–33. doi: 10.1161/circresaha.116.310293 PMC5546003 28607103

[104] Thorp EB. Mechanisms of failed apoptotic cell clearance by phagocyte subsets in cardiovascular disease. Apoptosis. (2010) 15:1124–36. doi: 10.1007/s10495-010-0516-6 PMC3744319 20552278

[105] Glinton KE Ma W Lantz C Grigoryeva LS DeBerge M Liu X. Macrophage-produced vegfc is induced by efferocytosis to ameliorate cardiac injury and inflammation. J Clin Invest. (2022) 132. doi: 10.1172/jci140685 PMC9057589 35271504

[106] Fond AM Lee CS Schulman IG Kiss RS Ravichandran KS. Apoptotic cells trigger a membrane-initiated pathway to increase abca1. J Clin Invest. (2015) 125:2748–58. doi: 10.1172/jci80300 PMC4563683 26075824

[107] Jia D Chen S Bai P Luo C Liu J Sun A. Cardiac resident macrophage-derived legumain improves cardiac repair by promoting clearance and degradation of apoptotic cardiomyocytes after myocardial infarction. Circulation. (2022) 145:1542–56. doi: 10.1161/circulationaha.121.057549 35430895

[108] Frasch SC McNamee EN Kominsky D Jedlicka P Jakubzick C Zemski Berry K. G2a signaling dampens colitic inflammation *via* production of ifn-Γ. J Immunol (Baltimore Md: 1950). (2016) 197:1425–34. doi: 10.4049/jimmunol.1600264 PMC4975950 27402702

[109] Tian L Choi SC Murakami Y Allen J Morse HC 3rd Qi CF. P85α Recruitment by the cd300f phosphatidylserine receptor mediates apoptotic cell clearance required for autoimmunity suppression. Nat Commun. (2014) 5:3146. doi: 10.1038/ncomms4146 24477292 PMC4151829

[110] Huveneers S Truong H Danen HJ. Integrins: signaling, disease, and therapy. Int J Radiat Biol. (2007) 83:743–51. doi: 10.1080/09553000701481808 17852562

[111] Koehn OJ Lorimer E Unger B Harris R Das AS Suazo KF. Gtpase splice variants rac1 and rac1b display isoform-specific differences in localization, prenylation, and interaction with the chaperone protein smggds. J Biol Chem. (2023) 299:104698. doi: 10.1016/j.jbc.2023.104698 37059183 PMC10206184

[112] Karunakaran U Elumalai S Moon JS Won KC. Cd36 signal transduction in metabolic diseases: novel insights and therapeutic targeting. Cells. (2021) 10. doi: 10.3390/cells10071833 PMC8305429 34360006

[113] Boada-Romero E Martinez J Heckmann BL Green DR. The clearance of dead cells by efferocytosis. Nat Rev Mol Cell Biol. (2020) 21:398–414. doi: 10.1038/s41580-020-0232-1 32251387 PMC7392086

[114] Das S Sarkar A Ryan KA Fox S Berger AH Juncadella IJ. Brain angiogenesis inhibitor 1 is expressed by gastric phagocytes during infection with helicobacter pylori and mediates the recognition and engulfment of human apoptotic gastric epithelial cells. FASEB J. (2014) 28:2214–24. doi: 10.1096/fj.13-243238 PMC3986834 24509909

[115] Bosurgi L Bernink JH Delgado Cuevas V Gagliani N Joannas L Schmid ET. Paradoxical role of the proto-oncogene axl and mer receptor tyrosine kinases in colon cancer. Proc Natl Acad Sci United States America. (2013) 110:13091–6. doi: 10.1073/pnas.1302507110 PMC3740859 23878224

[116] Martin-Rodriguez O Gauthier T Bonnefoy F Couturier M Daoui A Chagué C. Pro-resolving factors released by macrophages after efferocytosis promote mucosal wound healing in inflammatory bowel disease. Front Immunol. (2021) 12:754475. doi: 10.3389/fimmu.2021.754475 35003066 PMC8727348

[117] Baumann I Kolowos W Voll RE Manger B Gaipl U Neuhuber WL. Impaired uptake of apoptotic cells into tingible body macrophages in germinal centers of patients with systemic lupus erythematosus. Arthritis rheumatism. (2002) 46:191–201. doi: 10.1002/1529-0131(200201)46:1<191::Aid-art10027>3.0.Co;2-k 11817590

[118] Le LQ Kabarowski JH Weng Z Satterthwaite AB Harvill ET Jensen ER. Mice lacking the orphan G protein-coupled receptor G2a develop a late-onset autoimmune syndrome. Immunity. (2001) 14:561–71. doi: 10.1016/s1074-7613(01)00145-5 11371358

[119] Choi SC Simhadri VR Tian L Gil-Krzewska A Krzewski K Borrego F. Cutting edge: mouse cd300f (Cmrf-35-like molecule-1) recognizes outer membrane-exposed phosphatidylserine and can promote phagocytosis. J Immunol (Baltimore Md: 1950). (2011) 187:3483–7. doi: 10.4049/jimmunol.1101549 PMC3178745 21865548

[120] ten Kate MK van der Meer J. Protein S deficiency: A clinical perspective. Haemophilia. (2008) 14:1222–8. doi: 10.1111/j.1365-2516.2008.01775.x 18479427

[121] Patten DA. Scarf1: A multifaceted, yet largely understudied, scavenger receptor. Inflammation Res. (2018) 67:627–32. doi: 10.1007/s00011-018-1154-7 PMC6028831 29725698

[122] Mike EV Makinde HM Der E Stock A Gulinello M Gadhvi GT. Neuropsychiatric systemic lupus erythematosus is dependent on sphingosine-1-phosphate signaling. Front Immunol. (2018) 9:2189. doi: 10.3389/fimmu.2018.02189 30319641 PMC6168636

[123] Liu Y Chen H Chen Z Qiu J Pang H Zhou Z. Novel roles of the tim family in immune regulation and autoimmune diseases. Front Immunol. (2021) 12:748787. doi: 10.3389/fimmu.2021.748787 34603337 PMC8484753

[124] Yavuz S Pucholt P Sandling JK Bianchi M Leonard D Bolin K. Mer-tyrosine kinase: A novel susceptibility gene for sle related end-stage renal disease. Lupus Sci Med. (2022) 9. doi: 10.1136/lupus-2022-000752 PMC9639142 36332927

[125] Coss SL Zhou D Chua GT Aziz RA Hoffman RP Wu YL. The complement system and human autoimmune diseases. J Autoimmun. (2023) 137:102979. doi: 10.1016/j.jaut.2022.102979 36535812 PMC10276174

[126] Liu Y Luo S Zhan Y Wang J Zhao R Li Y. Increased expression of ppar-Γ Modulates monocytes into a M2-like phenotype in sle patients: an implicative protective mechanism and potential therapeutic strategy of systemic lupus erythematosus. Front Immunol. (2020) 11:579372. doi: 10.3389/fimmu.2020.579372 33584646 PMC7873911

[127] Zeng T Li SJ Ao W Zheng H Wu FX Chen Y. The detection of autoantibodies to atp-binding cassette transporter A1 and its role in the pathogenesis of atherosclerosis in patients with systemic lupus erythematosus. Clin Biochem. (2012) 45:1342–6. doi: 10.1016/j.clinbiochem.2012.06.009 22709931

[128] Orme JJ Du Y Vanarsa K Mayeux J Li L Mutwally A. Heightened cleavage of axl receptor tyrosine kinase by adam metalloproteases may contribute to disease pathogenesis in sle. Clin Immunol (Orlando Fla). (2016) 169:58–68. doi: 10.1016/j.clim.2016.05.011 PMC5193537 27237127

[129] Thorp E Vaisar T Subramanian M Mautner L Blobel C Tabas I. Shedding of the mer tyrosine kinase receptor is mediated by adam17 protein through a pathway involving reactive oxygen species, protein kinase Cδ, and P38 mitogen-activated protein kinase (Mapk). J Biol Chem. (2011) 286:33335–44. doi: 10.1074/jbc.M111.263020 PMC3190938 21828049

[130] Ballantine L Midgley A Harris D Richards E Burgess S Beresford MW. Increased soluble phagocytic receptors smer, styro3 and saxl and reduced phagocytosis in juvenile-onset systemic lupus erythematosus. Pediatr Rheumatol Online J. (2015) 13:10. doi: 10.1186/s12969-015-0007-y 25878564 PMC4397859

[131] Kruse K Janko C Urbonaviciute V Mierke CT Winkler TH Voll RE. Inefficient clearance of dying cells in patients with sle: anti-dsdna autoantibodies, mfg-E8, hmgb-1 and other players. Apoptosis: an Int J programmed Cell Death. (2010) 15:1098–113. doi: 10.1007/s10495-010-0478-8 20198437

[132] Hanayama R Miyasaka K Nakaya M Nagata S. Mfg-E8-dependent clearance of apoptotic cells, and autoimmunity caused by its failure. Curr Dir Autoimmun. (2006) 9:162–72. doi: 10.1159/000090780 16394660

[133] van Zoelen MA van der Poll T. Targeting rage in sepsis. Crit Care (London England). (2008) 12:103. doi: 10.1186/cc6187 PMC2374639 18254937

[134] Nienhuis HL Westra J Smit AJ Limburg PC Kallenberg CG Bijl M. Age and their receptor rage in systemic autoimmune diseases: an inflammation propagating factor contributing to accelerated atherosclerosis. Autoimmunity. (2009) 42:302–4. doi: 10.1080/08916930902831746 19811283

[135] Manganelli V Truglia S Capozzi A Alessandri C Riitano G Spinelli FR. Alarmin hmgb1 and soluble rage as new tools to evaluate the risk stratification in patients with the antiphospholipid syndrome. Front Immunol. (2019) 10:460. doi: 10.3389/fimmu.2019.00460 30923525 PMC6426766

[136] Burnier L Saller F Kadi L Brisset AC Sugamele R Baudino L. Gas6 deficiency in recipient mice of allogeneic transplantation alleviates hepatic graft-versus-host disease. Blood. (2010) 115:3390–7. doi: 10.1182/blood-2009-02-206920 PMC2858482 20139094

[137] Chazaud B. Inflammation and skeletal muscle regeneration: leave it to the macrophages! Trends Immunol. (2020) 41:481–92. doi: 10.1016/j.it.2020.04.006 32362490

[138] Chiappalupi S Sorci G Vukasinovic A Salvadori L Sagheddu R Coletti D. Targeting rage prevents muscle wasting and prolongs survival in cancer cachexia. J cachexia sarcopenia Muscle. (2020) 11:929–46. doi: 10.1002/jcsm.12561 PMC7432590 32159297

[139] Riuzzi F Sorci G Sagheddu R Chiappalupi S Salvadori L Donato R. Rage in the pathophysiology of skeletal muscle. J cachexia sarcopenia Muscle. (2018) 9:1213–34. doi: 10.1002/jcsm.12350 PMC6351676 30334619

[140] Olaoba OT Kadasah S Vetter SW Leclerc E. Rage signaling in melanoma tumors. Int J Mol Sci. (2020) 21. doi: 10.3390/ijms21238989 PMC7730603 33256110

[141] Wu G Ma Z Hu W Wang D Gong B Fan C. Molecular insights of gas6/tam in cancer development and therapy. Cell Death Dis. (2017) 8:e2700. doi: 10.1038/cddis.2017.113 28333143 PMC5386520

[142] Bossi F Tripodo C Rizzi L Bulla R Agostinis C Guarnotta C. C1q as a unique player in angiogenesis with therapeutic implication in wound healing. Proc Natl Acad Sci United States America. (2014) 111:4209–14. doi: 10.1073/pnas.1311968111 PMC3964125 24591625

[143] Kawane K Ohtani M Miwa K Kizawa T Kanbara Y Yoshioka Y. Chronic polyarthritis caused by mammalian DNA that escapes from degradation in macrophages. Nature. (2006) 443:998–1002. doi: 10.1038/nature05245 17066036

[144] Waterborg CEJ Beermann S Broeren MGA Bennink MB Koenders MI van Lent P. Protective role of the mer tyrosine kinase *via* efferocytosis in rheumatoid arthritis models. Front Immunol. (2018) 9:742. doi: 10.3389/fimmu.2018.00742 29706963 PMC5908888

[145] Marei H Malliri A. Rac1 in human diseases: the therapeutic potential of targeting rac1 signaling regulatory mechanisms. Small GTPases. (2017) 8:139–63. doi: 10.1080/21541248.2016.1211398 PMC5584733 27442895

[146] Dardenne C Salon M Authier H Meunier E AlaEddine M Bernad J. Topical Aspirin Administration Improves Cutaneous Wound Healing in Diabetic Mice through a Phenotypic Switch of Wound Macrophages toward an Anti-Inflammatory and Proresolutive Profile Characterized by Lxa4 Release. Diabetes. (2022) 71:2181–96. doi: 10.2337/db20-1245 35796692

[147] Sen R. Control of B lymphocyte apoptosis by the transcription factor nf-kappab. Immunity. (2006) 25:871–83. doi: 10.1016/j.immuni.2006.12.003 17174931

[148] Doering J Begue B Lentze MJ Rieux-Laucat F Goulet O Schmitz J. Induction of T lymphocyte apoptosis by sulphasalazine in patients with crohn's disease. Gut. (2004) 53:1632–8. doi: 10.1136/gut.2003.037911 PMC1774288 15479684

[149] Klichinsky M Ruella M Shestova O Lu XM Best A Zeeman M. Human chimeric antigen receptor macrophages for cancer immunotherapy. Nat Biotechnol. (2020) 38:947–53. doi: 10.1038/s41587-020-0462-y PMC7883632 32361713

[150] Wu Y Huang L Sai W Chen F Liu Y Han C. Hbsp improves kidney ischemia-reperfusion injury and promotes repair in properdin deficient mice *via* enhancing phagocytosis of tubular epithelial cells. Front Immunol. (2023) 14:1183768. doi: 10.3389/fimmu.2023.1183768 37207230 PMC10188997

[151] Morioka S Kajioka D Yamaoka Y Ellison RM Tufan T Werkman IL. Chimeric efferocytic receptors improve apoptotic cell clearance and alleviate inflammation. Cell. (2022) 185:4887–903.e17. doi: 10.1016/j.cell.2022.11.029 36563662 PMC9930200

[152] Cai B Thorp EB Doran AC Subramanian M Sansbury BE Lin CS. Mertk cleavage limits proresolving mediator biosynthesis and exacerbates tissue inflammation. Proc Natl Acad Sci United States America. (2016) 113:6526–31. doi: 10.1073/pnas.1524292113 PMC4988577 27199481

[153] Ulland TK Colonna M. Trem2 - a key player in microglial biology and alzheimer disease. Nat Rev Neurol. (2018) 14:667–75. doi: 10.1038/s41582-018-0072-1 30266932

[154] Katzenelenbogen Y Sheban F Yalin A Yofe I Svetlichnyy D Jaitin DA. Coupled scrna-seq and intracellular protein activity reveal an immunosuppressive role of trem2 in cancer. Cell. (2020) 182:872–85.e19. doi: 10.1016/j.cell.2020.06.032 32783915

[155] Molgora M Esaulova E Vermi W Hou J Chen Y Luo J. Trem2 modulation remodels the tumor myeloid landscape enhancing anti-pd-1 immunotherapy. Cell. (2020) 182:886–900.e17. doi: 10.1016/j.cell.2020.07.013 32783918 PMC7485282

[156] Gardai SJ McPhillips KA Frasch SC Janssen WJ Starefeldt A Murphy-Ullrich JE. Cell-surface calreticulin initiates clearance of viable or apoptotic cells through trans-activation of lrp on the phagocyte. Cell. (2005) 123:321–34. doi: 10.1016/j.cell.2005.08.032 16239148

[157] Hao Y Zhou X Li Y Li B Cheng L. The cd47-sirpα Axis is a promising target for cancer immunotherapies. Int Immunopharmacol. (2023) 120:110255. doi: 10.1016/j.intimp.2023.110255 37187126

[158] Son J Hsieh RC Lin HY Krause KJ Yuan Y Biter AB. Inhibition of the cd47-sirpα Axis for cancer therapy: A systematic review and meta-analysis of emerging clinical data. Front Immunol. (2022) 13:1027235. doi: 10.3389/fimmu.2022.1027235 36439116 PMC9691650

[159] Kauder SE Kuo TC Harrabi O Chen A Sangalang E Doyle L. Alx148 blocks cd47 and enhances innate and adaptive antitumor immunity with a favorable safety profile. PloS One. (2018) 13:e0201832. doi: 10.1371/journal.pone.0201832 30133535 PMC6104973

[160] Chen SH Dominik PK Stanfield J Ding S Yang W Kurd N. Dual checkpoint blockade of cd47 and pd-L1 using an affinity-tuned bispecific antibody maximizes antitumor immunity. J immunotherapy Cancer. (2021) 9. doi: 10.1136/jitc-2021-003464 PMC8488710 34599020

[161] Mehrotra P Ravichandran KS. Drugging the efferocytosis process: concepts and opportunities. Nat Rev Drug Discovery. (2022) 21:601–20. doi: 10.1038/s41573-022-00470-y PMC9157040 35650427

[162] Liu Y Huang Q He M Chen T Chu X. A nano-bioconjugate modified with anti-sirpα Antibodies and antisense oligonucleotides of mtor for anti-atherosclerosis therapy. Acta biomaterialia. (2023). doi: 10.1016/j.actbio.2023.12.031 38160854

[163] Harding JJ Moreno V Bang YJ Hong MH Patnaik A Trigo J. Blocking tim-3 in treatment-refractory advanced solid tumors: A phase ia/B study of ly3321367 with or without an anti-pd-L1 antibody. Clin Cancer Res. (2021) 27:2168–78. doi: 10.1158/1078-0432.Ccr-20-4405 33514524

[164] Tirado-Gonzalez I Descot A Soetopo D Nevmerzhitskaya A Schäffer A Kur IM. Axl inhibition in macrophages stimulates host-versus-leukemia immunity and eradicates naïve and treatment-resistant leukemia. Cancer Discovery. (2021) 11:2924–43. doi: 10.1158/2159-8290.Cd-20-1378 PMC7611942 34103328

[165] Jeon JY Buelow DR Garrison DA Niu M Eisenmann ED Huang KM. Tp-0903 is active in models of drug-resistant acute myeloid leukemia. JCI Insight. (2020) 5. doi: 10.1172/jci.insight.140169 PMC7714403 33268594

[166] Zhu C Wei Y Wei X. Axl receptor tyrosine kinase as a promising anti-cancer approach: functions, molecular mechanisms and clinical applications. Mol Cancer. (2019) 18:153. doi: 10.1186/s12943-019-1090-3 31684958 PMC6827209

[167] Tang Y Zang H Wen Q Fan S. Axl in cancer: A modulator of drug resistance and therapeutic target. J Exp Clin Cancer research: CR. (2023) 42:148. doi: 10.1186/s13046-023-02726-w 37328828 PMC10273696

[168] Stanford JC Young C Hicks D Owens P Williams A Vaught DB. Efferocytosis produces a prometastatic landscape during postpartum mammary gland involution. J Clin Invest. (2014) 124:4737–52. doi: 10.1172/jci76375 PMC4347249 25250573

[169] Zhou Y Fei M Zhang G Liang WC Lin W Wu Y. Blockade of the phagocytic receptor mertk on tumor-associated macrophages enhances P2x7r-dependent sting activation by tumor-derived cgamp. Immunity. (2020) 52:357–73.e9. doi: 10.1016/j.immuni.2020.01.014 32049051

[170] Du W Zhu J Zeng Y Liu T Zhang Y Cai T. Kpnb1-mediated nuclear translocation of pd-L1 promotes non-small cell lung cancer cell proliferation *via* the gas6/mertk signaling pathway. Cell Death differentiation. (2021) 28:1284–300. doi: 10.1038/s41418-020-00651-5 PMC8027631 33139930

[171] Myers KV Amend SR Pienta KJ. Targeting tyro3, axl and mertk (Tam receptors): implications for macrophages in the tumor microenvironment. Mol Cancer. (2019) 18:94. doi: 10.1186/s12943-019-1022-2 31088471 PMC6515593

[172] Sang YB Kim JH Kim CG Hong MH Kim HR Cho BC. The development of axl inhibitors in lung cancer: recent progress and challenges. Front Oncol. (2022) 12:811247. doi: 10.3389/fonc.2022.811247 35311091 PMC8927964

[173] Zhou X Li D Xia S Ma X Li R Mu Y. Rna-based modulation of macrophage-mediated efferocytosis potentiates antitumor immunity in colorectal cancer. J Controlled release. (2023) 366:128–41. doi: 10.1016/j.jconrel.2023.12.018 38104775

[174] Bruemmer NC Holland JF Sheehe PR. Drug effects on a target metabolic pathway and on mouse tumor growth: azauridine and decarboxylation of orotic acid-7-C-14. Cancer Res. (1962) 22:113–9. 13873893

[175] Greggio C Jha P Kulkarni SS Lagarrigue S Broskey NT Boutant M. Enhanced respiratory chain supercomplex formation in response to exercise in human skeletal muscle. Cell Metab. (2017) 25:301–11. doi: 10.1016/j.cmet.2016.11.004 27916530

[176] Hammel P Fabienne P Mineur L Metges JP Andre T de la Fouchardiere C. Erythrocyte-encapsulated asparaginase (Eryaspase) combined with chemotherapy in second-line treatment of advanced pancreatic cancer: an open-label, randomized phase iib trial. Eur J Cancer (Oxford England: 1990). (2020) 124:91–101. doi: 10.1016/j.ejca.2019.10.020 31760314

[177] Yulian ED Siregar NC Sudijono B Hwei LRY. The role of hmgcr expression in combination therapy of simvastatin and fac treated locally advanced breast cancer patients. Breast Dis. (2023) 42:73–83. doi: 10.3233/bd-220021 36938720

[178] Shi D Wu X Jian Y Wang J Huang C Mo S. Usp14 promotes tryptophan metabolism and immune suppression by stabilizing ido1 in colorectal cancer. Nat Commun. (2022) 13:5644. doi: 10.1038/s41467-022-33285-x 36163134 PMC9513055

[179] Zuniga KE Parma DL Muñoz E Spaniol M Wargovich M Ramirez AG. Dietary intervention among breast cancer survivors increased adherence to a mediterranean-style, anti-inflammatory dietary pattern: the rx for better breast health randomized controlled trial. Breast Cancer Res Treat. (2019) 173:145–54. doi: 10.1007/s10549-018-4982-9 PMC6387648 30259284

[180] Xiao Y Yu TJ Xu Y Ding R Wang YP Jiang YZ. Emerging therapies in cancer metabolism. Cell Metab. (2023) 35:1283–303. doi: 10.1016/j.cmet.2023.07.006 37557070

[181] Raymond MH Davidson AJ Shen Y Tudor DR Lucas CD Morioka S. Live cell tracking of macrophage efferocytosis during drosophila embryo development in vivo. Sci (New York NY). (2022) 375:1182–7. doi: 10.1126/science.abl4430 PMC7612538 35271315

[182] Batoon L Koh AJ Kannan R McCauley LK Roca H. Caspase-9 driven murine model of selective cell apoptosis and efferocytosis. Cell Death Dis. (2023) 14:58. doi: 10.1038/s41419-023-05594-6 36693838 PMC9873735

[183] Shi J Wu X Wang Z Li F Meng Y Moore RM. A genome-wide crispr screen identifies wdfy3 as a regulator of macrophage efferocytosis. Nat Commun. (2022) 13:7929. doi: 10.1038/s41467-022-35604-8 36566259 PMC9789999

[184] Chandel NS. Carbohydrate metabolism. Cold Spring Harbor Perspect Biol. (2021) 13. doi: 10.1101/cshperspect.a040568 PMC7778149 33397651

[185] Pan Y Yu Y Wang X Zhang T. Tumor-associated macrophages in tumor immunity. Front Immunol. (2020) 11:583084. doi: 10.3389/fimmu.2020.583084 33365025 PMC7751482

[186] Schilperoort M Ngai D Katerelos M Power DA Tabas I. Pfkfb2-mediated glycolysis promotes lactate-driven continual efferocytosis by macrophages. Nat Metab. (2023) 5:431–44. doi: 10.1038/s42255-023-00736-8 PMC10050103 36797420

[187] Manoharan I Prasad PD Thangaraju M Manicassamy S. Lactate-dependent regulation of immune responses by dendritic cells and macrophages. Front Immunol. (2021) 12:691134. doi: 10.3389/fimmu.2021.691134 34394085 PMC8358770

[188] Chen P Zuo H Xiong H Kolar MJ Chu Q Saghatelian A. Gpr132 sensing of lactate mediates tumor-macrophage interplay to promote breast cancer metastasis. Proc Natl Acad Sci United States America. (2017) 114:580–5. doi: 10.1073/pnas.1614035114 PMC5255630 28049847

[189] Herzig S Shaw RJ. Ampk: guardian of metabolism and mitochondrial homeostasis. Nat Rev Mol Cell Biol. (2018) 19:121–35. doi: 10.1038/nrm.2017.95 PMC5780224 28974774

[190] Ngai D Schilperoort M Tabas I. Efferocytosis-induced lactate enables the proliferation of pro-resolving macrophages to mediate tissue repair. Nat Metab. (2023) 5:2206–19. doi: 10.1038/s42255-023-00921-9 PMC10782856 38012414

[191] Song W Li D Tao L Luo Q Chen L. Solute carrier transporters: the metabolic gatekeepers of immune cells. Acta Pharm Sin B. (2020) 10:61–78. doi: 10.1016/j.apsb.2019.12.006 31993307 PMC6977534

[192] He D Mao Q Jia J Wang Z Liu Y Liu T. Pentose phosphate pathway regulates tolerogenic apoptotic cell clearance and immune tolerance. Front Immunol. (2021) 12:797091. doi: 10.3389/fimmu.2021.797091 35082786 PMC8784392

[193] Wang YT Trzeciak AJ Rojas WS Saavedra P Chen YT Chirayil R. Metabolic adaptation supports enhanced macrophage efferocytosis in limited-oxygen environments. Cell Metab. (2023) 35:316–31.e6. doi: 10.1016/j.cmet.2022.12.005 36584675 PMC9908853

[194] Madenspacher JH Morrell ED Gowdy KM McDonald JG Thompson BM Muse G. Cholesterol 25-hydroxylase promotes efferocytosis and resolution of lung inflammation. JCI Insight. (2020) 5. doi: 10.1172/jci.insight.137189 PMC7308063 32343675

[195] Chen L Zhao ZW Zeng PH Zhou YJ Yin WJ. Molecular mechanisms for abca1-mediated cholesterol efflux. Cell Cycle (Georgetown Tex). (2022) 21:1121–39. doi: 10.1080/15384101.2022.2042777 PMC9103275 35192423

[196] Barreda D Grinstein S Freeman SA. Target lysis by cholesterol extraction is a rate limiting step in the resolution of phagolysosomes. Eur J Cell Biol. (2023) 103:151382. doi: 10.1016/j.ejcb.2023.151382 38171214

[197] Körner A Schlegel M Theurer J Frohnmeyer H Adolph M Heijink M. Resolution of inflammation and sepsis survival are improved by dietary Ω-3 fatty acids. Cell Death differentiation. (2018) 25:421–31. doi: 10.1038/cdd.2017.177 PMC5762854 29053142

[198] Quiros M Nusrat A. Saving problematic mucosae: spms in intestinal mucosal inflammation and repair. Trends Mol Med. (2019) 25:124–35. doi: 10.1016/j.molmed.2018.12.004 30642681

[199] Koenis DS de Matteis R Rajeeve V Cutillas P Dalli J. Efferocyte-derived mctrs metabolically prime macrophages for continual efferocytosis *via* rac1-mediated activation of glycolysis. Advanced Sci (Weinheim Baden-Wurttemberg Germany). (2023):e2304690. doi: 10.1002/advs.202304690 PMC10870015 38064171

[200] Merlin J Ivanov S Dumont A Sergushichev A Gall J Stunault M. Non-canonical glutamine transamination sustains efferocytosis by coupling redox buffering to oxidative phosphorylation. Nat Metab. (2021) 3:1313–26. doi: 10.1038/s42255-021-00471-y PMC7611882 34650273

[201] McCubbrey AL McManus SA McClendon JD Thomas SM Chatwin HB Reisz JA. Polyamine import and accumulation causes immunomodulation in macrophages engulfing apoptotic cells. Cell Rep. (2022) 38:110222. doi: 10.1016/j.celrep.2021.110222 35021097 PMC8859864

[202] Yang L Zheng C Xia YF Dai Y Wei ZF. 3, 3'-diindolylmethane enhances macrophage efferocytosis and subsequently relieves visceral pain *via* the ahr/nrf2/arg-1-mediated arginine metabolism pathway. Phytomedicine. (2023) 116:154874. doi: 10.1016/j.phymed.2023.154874 37216760

[203] Cheng L Weng B Jia C Zhang L Hu B Deng L. The expression and significance of efferocytosis and immune checkpoint related molecules in pancancer samples and the correlation of their expression with anticancer drug sensitivity. Front Pharmacol. (2022) 13:977025. doi: 10.3389/fphar.2022.977025 36059952 PMC9437300

[204] Werfel TA Elion DL Rahman B Hicks DJ Sanchez V Gonzales-Ericsson PI. Treatment-induced tumor cell apoptosis and secondary necrosis drive tumor progression in the residual tumor microenvironment through mertk and ido1. Cancer Res. (2019) 79:171–82. doi: 10.1158/0008-5472.Can-18-1106 30413412

[205] Ampomah PB Cai B Sukka SR Gerlach BD Yurdagul A Jr. Wang X. Macrophages use apoptotic cell-derived methionine and dnmt3a during efferocytosis to promote tissue resolution. Nat Metab. (2022) 4:444–57. doi: 10.1038/s42255-022-00551-7 PMC9050866 35361955

[206] Wu Y Wang C Yan Y Hao Y Liu B Dong Z. Efferocytosis nanoinhibitors to promote secondary necrosis and potentiate the immunogenicity of conventional cancer therapies for improved therapeutic benefits. ACS nano. (2023) 17:18089–102. doi: 10.1021/acsnano.3c04884 37669546

